# Enhanced Generalizability of RNA Secondary Structure Prediction via Convolutional Block Attention Network and Ensemble Learning

**DOI:** 10.3390/molecules30163447

**Published:** 2025-08-21

**Authors:** Hanbo Lin, Dongyue Hou, Zhaoyite Li, Shuaiqi Wang, Yuchen Liu, Jiajie Gu, Juncheng Qian, Ruining Yin, Hui Zhao, Shaofei Wang, Yuzong Chen, Dianwen Ju, Xian Zeng

**Affiliations:** 1 School of Pharmaceutical Sciences, Shanghai Engineering Research Center of Immunotherapeutics, Fudan University, Shanghai 201203, China; hblin23@m.fudan.edu.cn (H.L.); dyhou22@m.fudan.edu.cn (D.H.); wangsq20@fudan.edu.cn (S.W.); liuyc21@m.fudan.edu.cn (Y.L.); jjgu21@m.fudan.edu.cn (J.G.); jcqian21@m.fudan.edu.cn (J.Q.); rnyin24@m.fudan.edu.cn (R.Y.); 2 The State Key Laboratory of Chemical Oncogenomics, Key Laboratory of Chemical Biology, Tsinghua Shenzhen International Graduate School, Tsinghua University, Shenzhen 518055, China; lizhaoyite@gmail.com; 3 Byterna Therapeutics Ltd., Shanghai 201203, China; zhaohui@byterna.com; 4 Department of Cellular and Genetic Medicine, School of Basic Medical Sciences, Fudan University, Shanghai 200032, China; shaofeiwang@fudan.edu.cn; 5 Institute of Biomedical Health Technology and Engineering, Shenzhen Bay Laboratory, Shenzhen 518000, China

**Keywords:** RNA secondary structure prediction, ensemble learning, convolutional block attention network

## Abstract

The determination of RNA secondary structure (RSS) could help understand RNA’s functional mechanisms, guiding the design of RNA-based therapeutics, and advancing synthetic biology applications. However, traditional methods such as NMR for determining RSS are typically time-consuming and labor-intensive. As a result, the accurate prediction of RSS remains a fundamental yet unmet need in RNA research. Various deep learning (DL)-based methods achieved improved accuracy over thermodynamic-based methods. However, the over-parameterization nature of DL makes these methods prone to overfitting and thus limits their generalizability. Meanwhile, the inconsistency of RSS predictions between these methods further aggravated the crisis of generalizability. Here, we propose TrioFold to achieve enhanced generalizability of RSS prediction by integrating base-pairing clues learned from both thermodynamic- and DL-based methods by ensemble learning and convolutional block attention mechanism. TrioFold achieves higher accuracy in intra-family predictions and enhanced generalizability in inter-family and cross-RNA-types predictions. Additionally, we have developed an online webserver equipped with widely used RSS prediction algorithms and analysis tools, providing an accessible platform for the RNA research community. This study demonstrated new opportunities to improve generalizability for RSS predictions by efficient ensemble learning of base-pairing clues learned from both thermodynamic- and DL-based algorithms.

## 1. Introduction

RNA finds extensive applications in biology, medicine, synthetic biology, and other functional purposes, in which the RNA secondary structure (RSS) is fundamental to carrying diverse functionalities [1,2]. Characterization of RSS is crucial for understanding and designing functional RNA molecules. For example, a comprehensive analysis of RSS yields essential insights into gene transcription, catalytic activity, and RNA stability under diverse conditions [3], which helps understand the biological functions of RNA and the mechanism of related diseases. Accurately predicting RSS is also the key step in RNA molecule design using generative artificial intelligence (AI) algorithms [4]. Traditional biological experiments usually rely on magnetic resonance imaging, X-ray crystallization, enzyme probing, and other methods [5,6] to determine the RSS. However, these methods exhibit protracted experimental durations and incur high experimental costs. Additionally, the inherent instability of RNA molecules and the challenges of in vitro crystallization make it more difficult to carry out related experiments. Only less than 0.001% of the non-coding RNAs have been experimentally determined [7].

Given the time-consuming and expensive nature of traditional methods for determining RSS, there is an imperative to develop efficient and accurate RSS prediction algorithms, which has become a focal concern in RNA research. In recent years, machine learning (ML)- and deep learning (DL)-based algorithms have gained substantial attention and achieved significant advancements in structural biology [8,9]. Notably, the field has witnessed increased interest in developing RSS prediction algorithms based on ML and DL. For example, some ML algorithms such as UFold [10], RNAformer [11], RNAErnie [12], and RNADiffFold [13] reported in recent years have introduced novel tools for RSS prediction with enhanced prediction accuracy. In addition, online tools such as RNAthor [14] and RNAProbe [15] provide user-friendly platforms showcasing advanced methods for integrating experimental probing results with computational RNA structure prediction, which offer new insights into the RSS prediction field.

Although these ML/DL-based algorithms have shown improved prediction accuracy over classical thermodynamic-based methods like RNAfold [16] and RNAstructure [17], the existing methods suffer several limitations [18]. Firstly, the prediction accuracy of individual methods varied across RNA families. Secondly, there is a low concordance of the prediction results between individual methods for the same RNAs. This may stem from the fact that each algorithm only captured a partial of RNA folding rules from different perspectives. The limited generalizability of current algorithms is the fundamental problem underlying the above-mentioned limitations. Therefore, there is a strong need for novel methods with improved generalizability to advance deep learning-based RSS algorithms.

Different algorithms use different underlying principles, so their predictions are prone to being less consistent. However, each type of method captures the rules of RNA folding to some extent [19]. Therefore, we hypothesize that the ensemble of algorithms that learn from diverse RNA folding principles will improve both prediction accuracy and generalizability.

Ensemble learning, including bagging, boosting, and stacking strategies, integrates the predictions of multiple individual models, known as base learners, to enhance overall predictive performance and increase generalizability. Ensemble learning can alleviate output biases arising from diverse base learners, thus improving the generalizability of models [20]. In the context of training with limited sample data [21], ensemble learning mitigates the overfitting problem in deep learning, consequently enhancing the generalizability and performance of the model [22].

**Figure 1:**
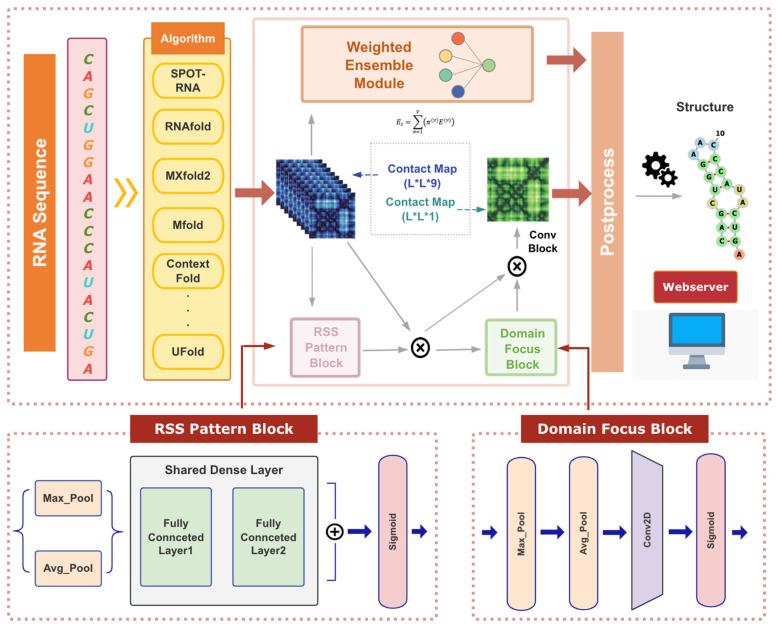
The network illustration of the TrioFold and TrioFold-lite.

Here, we proposed an end-to-end RSS prediction algorithm, TrioFold, based on ensemble deep learning and convolutional attention mechanism [23] (Figure 1). In detail, six representative ML models (MXfold2 [24], UFold [10], SPOT-RNA [25], EternaFold [26], CONTRAfold [27], and ContextFold [28]) and three thermodynamic-based models (Mfold [29], LinearFold [30], and RNAfold) were used as base learners to explore the impacts of different ensemble methods on RSS prediction accuracy and generalizability. Firstly, two different ensemble models were evaluated in the TestSetA and TS0 datasets. In the TestSetA and TS0 datasets, the F1 score of the TrioFold was 3–5% higher than that of the second-best model, and in other datasets, TrioFold showed strong prediction robustness. Furthermore, TrioFold achieved the best performance on three datasets of unseen families, suggesting its enhanced generalizability for new RNA sequences and families. Moreover, we implemented TrioFold and those base learner methods in a one-stop user-friendly webserver to enable convenient usage for biologists without any programming requirement. The webserver provides RSS prediction and analysis functions, and it can be freely accessed at http://triofold.aiddlab.com/ (accessed on 16 August 2025).

## 2. Results

### 2.1. Varied Outputs Among RSS Prediction Algorithms Despite Comparable Accuracies

**Figure 2:**
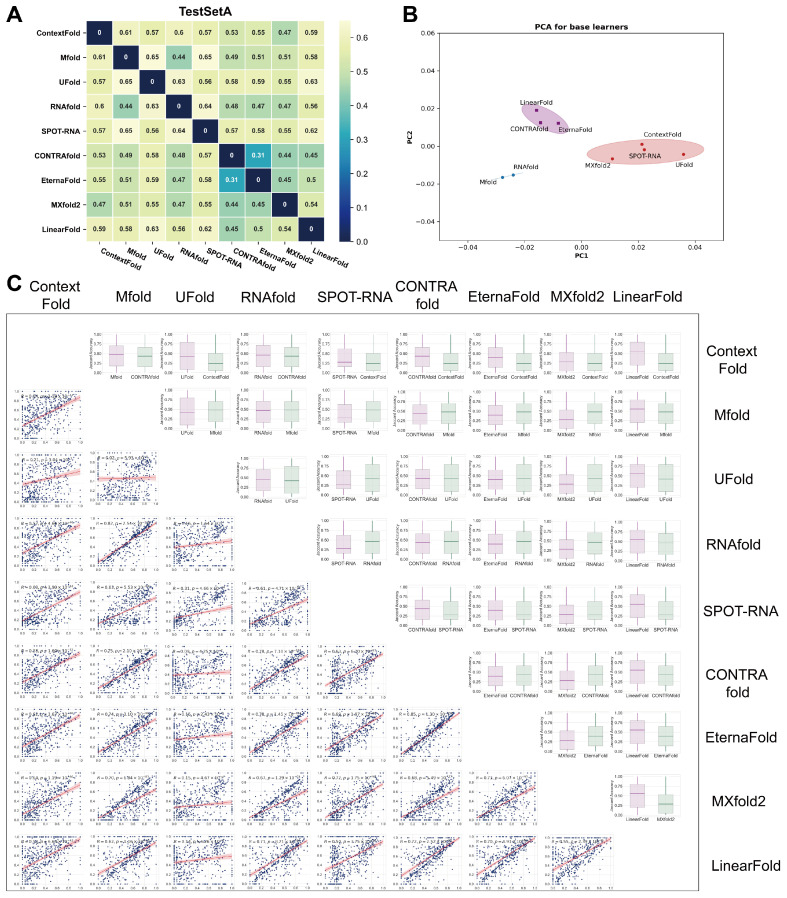
Significant differences in the output structures between different algorithms. Base learners’ concordance and prediction performance of ensemble models using different base learner combinations on TestSetA. (**A**) The Jaccard distance of RSS predicted by each base learner. The larger the Jaccard distance, the greater the difference in the predicted SS of the two base learners. (**B**) PCA results for base learners with ellipses. (**C**) The box plot illustrates the difference in accuracy of Jaccard distance between the two algorithms, while the scatter plot shows the correlation of Jaccard distance for all samples between algorithm pairs.

Although RSS prediction accuracy did not vary sharply across different algorithms, the concordance of their predictions was merely investigated. We attempted to tackle this question by comparing RSS predictions with nine commonly used algorithms. To measure the dissimilarity between the output of various predictive algorithms and the ground truth structure, we defined the Jaccard distance between the predicted structure and the ground truth structure on the same sequence as a metric. On the TestSetA dataset, for every two algorithms, the contact map of all samples in TestSetA was first predicted by each algorithm, and the average of Jaccard distances of contact maps of all samples was then calculated to represent the Jaccard distances of predictions between the two algorithms. The Jaccard distance between nine algorithms varies within the range of 0.3–0.65 (Figure 2A). The two algorithms with the greatest concordance are EternaFold and CONTRAfold (Jaccard distance = 0.31), which may result from the fact that EternaFold is an algorithm developed based on CONTRAfold. To investigate the distinctions among various algorithms, we employed PCA for dimensionality reduction (see Materials and Methods). PCA results also demonstrate the discordance between algorithms (Figure 2B). Unexpectedly, this analysis indicated that the output similarity of RSS predictions between different algorithms is usually low. A detailed visualization of the pair-wise comparison indicated that the accuracy of Jaccard distances between two algorithms only exhibited a weak degree of correlation in most cases (Figure 2C), further proving the diversity of different algorithms in RSS prediction.

### 2.2. Ensemble Learning Is Efficient in Enhancing RSS Prediction Performance

**Figure 3:**
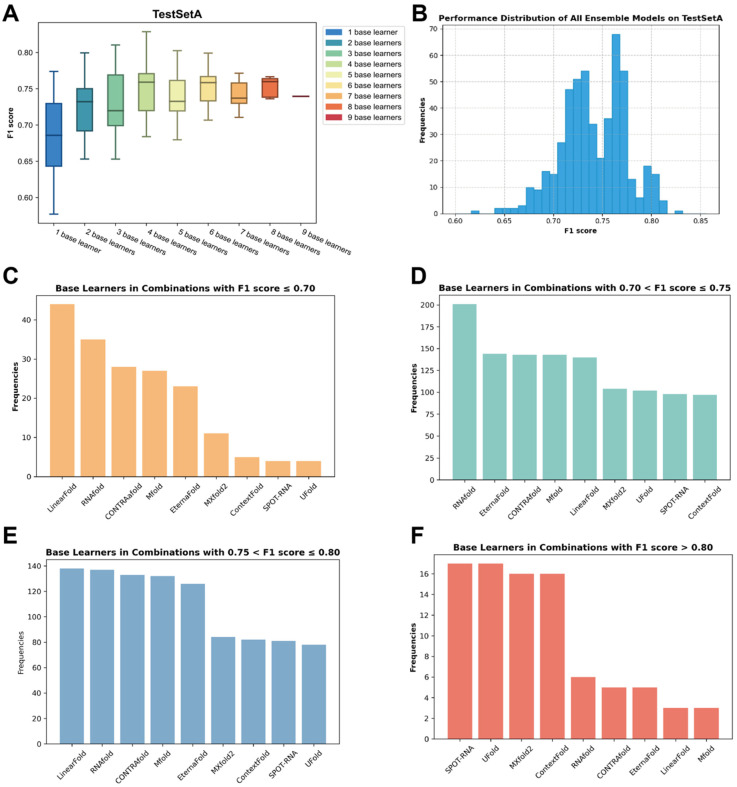
The impact of different ensemble strategies on the performance of the final model. (**A**) The impact of the ensemble size on the performance of the ensemble model. (**B**) The F1 score distribution of ensemble models based on all possible combinations on TestSetA. (**C**–**F**) The number of base learners appeared in all possible combinations according to the F1 score range.

Though ensemble models can be characterized into homogeneous ensembles or heterogeneous ensembles [31], the key to a successful ensemble model is the diversity of base learners. A crucial concept in ensemble methods is determining the optimal number of base learners to be included in the final ensemble, commonly referred to as the ensemble size [32]. However, the current knowledge on the diversity of RSS prediction base learners and their contributions to ensemble models is poor. To determine what combination of ensemble methods can improve the model’s performance to the greatest extent, we used TestSetA to test the impact of different ensemble methods on the model’s prediction performance. Nine base learners were selected as the candidate models. We systematically explored all possible combinations of base learners on the TestSetA, employing the F1 score as the benchmark metric for evaluating different combinations. As the number of base learners increases, the performance of the ensemble model does not exhibit a strictly monotonous upward trend but gradually converges toward a fairly stable value (Figure 3A) and the effects of different combinations vary considerably (Figure 3B). Notably, an optimal performing model emerges when the ensemble size is set at four. The ensemble model based on SPOT-RNA + UFold + MXfold2 + ContextFold (TrioFold-lite) performs best. Therefore, we chose an ensemble model based on the above four algorithms and conducted our following experiments.

Furthermore, in our observation of combinations exhibiting different F1 scores, we noted variations in the frequency of appearance of base learners (Table S1). We found that combinations with lower F1 scores demonstrate a higher occurrence of base learners such as RNAfold and Mfold. Conversely, in combinations yielding higher F1 scores, algorithms like UFold and ContextFold exhibit a higher frequency (Figure 3C–F). This signifies that the performance of base learners is a pivotal determinant of the overall performance of the ensemble model.

### 2.3. TrioFold Outperforms RSS Prediction Methods on Intra-Family Datasets

**Figure 4:**
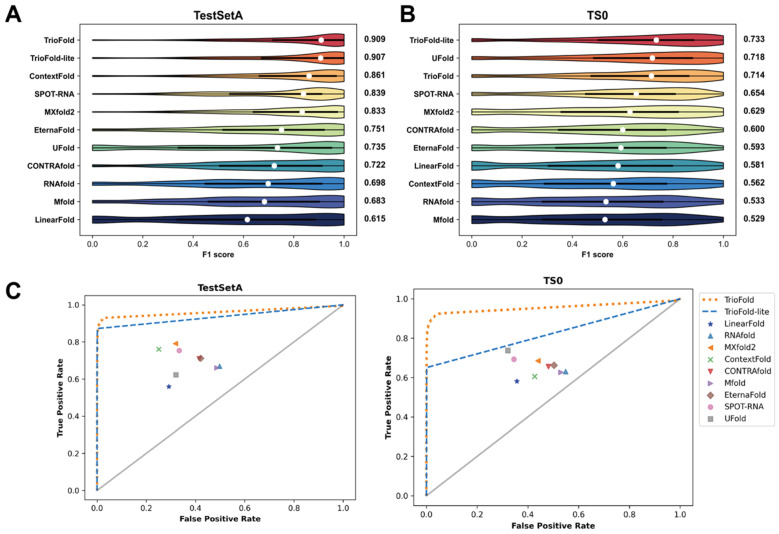
Performance comparison between TrioFold and existing methods. Distribution of F1 scores for different algorithms on TestSetA (**A**) and TS0 (**B**), respectively. (**C**) ROC curves on TestSetA and TS0 by various methods.

We employed two integration strategies, TrioFold and TrioFold-lite, for our investigations. See Methods for the detailed network architecture and setup. The RSS prediction performance of TrioFold and representative state-of-the-art (SOTA) methods was evaluated on two well-established benchmark datasets, TestSetA and TS0. Sequence identity analysis reveals that most sequences in TestSetA and TS0 share low similarity (<30% identity) with those in the training set (Tables S2 and S3). TrioFold and TrioFold-lite achieve a median F1 score of 0.909 and 0.907, respectively, which shows a notable improvement of 5.6% and 5.3%, respectively, when compared to the second-best model (Figure 4A). Among compared methods, the median F1 score of TrioFold is 23.7%, 8.34%, 5.57%, and 9.12% higher than the average F1 score of four base learners (UFold, SPOT-RNA, ContextFold, and MXfold2, respectively), indicating that the ensemble learning mechanisms of TrioFold are efficient. The median F1 score of TrioFold is 25.9%, 33.1%, 30.2%, 47.8%, and 21.0% higher than the median F1 score of the remaining methods (CONTRAfold, Mfold, RNAfold, LinearFold, and EternaFold, respectively). On the TestSetA, TrioFold-lite exhibits similar performance compared to TrioFold. The average computational time is also calculated (Table S4). On the TS0 dataset, TrioFold and TrioFold-lite achieve a median F1 score of 0.714 and 0.733, respectively. Here, TrioFold-lite achieves the best performance and TrioFold is slightly inferior to UFold. TrioFold-lite shows improvements in F1 scores ranging from 2.1% to 38.6% over SOTA methods (Figure 4B). The ROC curves also suggest the superior performance of TrioFold on TestSetA and TS0 datasets (Figure 4C). In addition, the results also show significant variations in different methods’ performance across the two datasets. For instance, SPOT-RNA achieves an F1 score of 0.839 on TestSetA, whereas it only achieves 0.654 on the TS0 dataset. This discrepancy may suggest that algorithms exhibit preferences when predicting sequences from different RNA types [33]. Together, these results indicated that the ensemble learning mechanisms of TrioFold are efficient in enhancing the RSS prediction performance.

### 2.4. Performance Comparison Between Algorithms Across RNA Types

RNA types categorize RNA molecules by their general functional roles, while RNA families group sequences within these types based on conserved sequence and structural features, reflecting evolutionary relationships and functional similarities. For RNA families that have appeared in the training set, deep learning models tend to have a good performance. Conversely, when dealing with RNA sequences from families not represented in the training set, the prediction accuracy tends to decline sharply.

**Figure 5:**
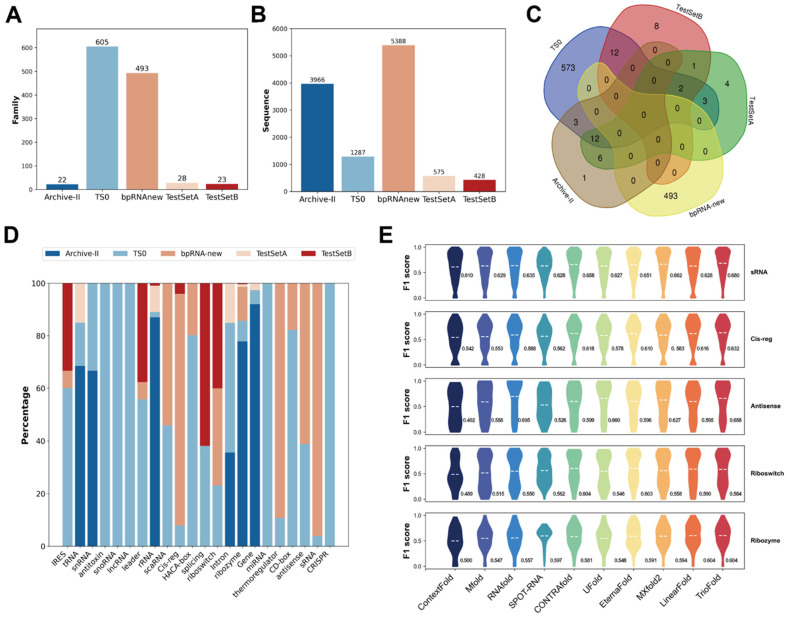
RNA SS prediction performance among different RNA clans. (**A**) Bar chart shows the distribution of Rfam families in certain datasets. (**B**) Bar chart shows the sequence numbers in certain datasets. (**C**) Venn chart shows the overlapping of different Rfam families in different datasets. (**D**) Histogram plot shows the distribution of RNA types on the TestSetA + TS0 + bpRNA-new + Archive-II. (**E**) Violin plot shows the performance on top-5 RNA CLANs on bpRNA-new.

To examine the distribution of RNA families in various datasets, we use Infernal 1.1.4 [34] software to map RNA sequences to specific families. The result shows that the distribution of RNA families in different datasets is extremely uneven (Figure 5A,C), and the number of sequences in certain datasets also varies (Figure 5B). The imbalanced distribution of RNA types in the dataset suggests potential variations in the performance of the model across different RNA types.

Due to the extensive number of RNA families, the dataset for each family is insufficient to support statistical analysis. A Rfam clan is a collection of families that either share a common ancestor but have diverged too significantly to be aligned reliably, or a group of families that could be aligned but possess distinct functions. When the family distribution is uneven, the distribution of clans in specific datasets is also uneven (Figure 5D). To evaluate prediction performance across various RNA clans, we first map the sequences from the RNA family to the clans, and choose the bpRNA-new dataset taking into account both sequence counts and clan distributions. TrioFold demonstrates robustness and accuracy across various RNA clans (Figure 5E).

### 2.5. The Generalizability of TrioFold to Predict RSS in Unseen Families

The RNA family that does not appear in the training set is called the unseen RNA family, and the RNA family that appears in the training set is called the intra-family RNA family. The main challenge in RSS prediction using deep learning is the prediction of unseen family sequences. This challenge arises from the limited sample size of existing RNA data and the imbalanced distribution of RNA types. The consequence is that deep learning models are prone to overlearning specific features of certain RNA types, resulting in overfitting and suboptimal performance when applied to unseen family sequences. The ensemble model’s ability to diminish variance between models and improve the deep learning model’s generalizability across unknown RNA types is a key benefit. Here, we used TS-inter, bpRNA-new, and a PDB-derived dataset [35] (see Materials and Methods) as unseen family datasets to determine the models’ generalizability on unseen sequences. Note that we have ensured that the aforementioned sequences were not included in the training set of any RNA prediction algorithm, thereby avoiding potential biases in assessing the model’s performance. Table 1, Table 2 and Table 3 show that the TrioFold achieves the best performance on all three datasets. To assess the statistical significance of these improvements, per-sequence paired *t*-tests comparing TrioFold against each baseline algorithm are also shown in the Tables.

Here, we compared the TrioFold with both DL-based and thermodynamic-based algorithms. TS-inter comprises sequences that are part of the TS0 dataset but have low similarity with the training sets. Consequently, these sequences can be regarded as unseen family sequences. Though UFold outperforms all other models on the TS0 dataset, its performance dropped drastically on unseen sequences in TS0, only achieving a performance similar to algorithms like RNAfold and MXfold2. Additionally, we categorized the TS-inter sequence structures based on their dissimilarity to the training set structures (calculated using RNAforester) as cutoffs. We recorded the performance of different models on these sequences (Figure S1).

To further benchmark TrioFold’s generalizability, we extended our evaluation to include the bpRNA-new and PDB datasets. To ensure that the model’s performance is not the result of data leakage, we used VSEARCH [36] to compare every sequence in these two datasets against the training set. The results show that 96.27% of sequences in bpRNA-new and 80.67% of those in PDB have less than 30% sequence identity to any training sequence (Tables S5 and S6). This low number indicates the independence of the training and test datasets. We also found that, on the bpRNA-new dataset, the DL-based model is inferior to those thermodynamic-based methods represented by LinearFold (Table 1). Moreover, we further conducted pseudoknot prediction evaluation on PDB datasets. Although most base learners are restricted to pseudoknot-free structures, our model exhibited limited but non-zero ability to capture pseudoknotted base pairs (Table S7), suggesting potential for future improvement.

In addition, we re-evaluated the concordance of RSS predictions of different algorithms including TrioFold and TrioFold-lite on TestSetA, as previously performed in Figure 2A. TrioFold and TrioFold-lite achieved the best RSS prediction accuracies (Figure 4A) and exhibited the lowest Jaccard distances to the remaining algorithms (0.19–0.53 and 0.19–0.57, respectively) (Figures S2 and S3). TrioFold and TrioFold-lite shown the lowest median of Jaccard distances to other algorithms (0.427 and 0.414, respectively), while SPOT-RNA (the median is 0.574, the range is 0.391–0.65) and UFold (the median is 0.569, the range is 0.296–0.649) had high Jaccard distances to other algorithms.

Collectively, our findings revealed that TrioFold achieves the best performance on unseen family datasets and enhanced concordance of RSS predictions among algorithms, further demonstrating the robustness and generalizability of the model to RSS prediction tasks. Additionally, TrioFold and TrioFold-lite models had a low number of trainable parameters compared with other algorithms (Table S8).

### 2.6. Showcase of SS Prediction on Representative RNAs from Unseen Families

**Figure 6:**
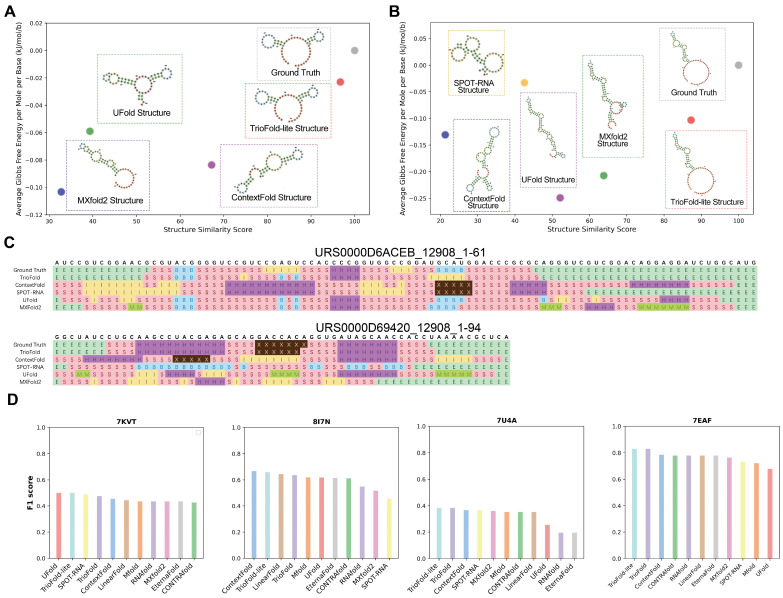
Showcases with representative examples from bpRNA-new and PDB. The RSS of (**A**) URS0000D6ACEB_12908_1-61 and (**B**) URS0000D69420_12908_1-94 were predicted by TrioFold, TrioFold-lite, and four base learners. A higher structure similarity indicates a greater similarity in SSs. In both cases, the TrioFold-lite predicted SS is more consistent with the ground truth structures. (**C**) Structure alignment for URS0000D6ACEB_12908_1-61 and URS0000D69420_12908_1-94, motif annotation is generated by bpRNA. Different letters represent different motifs. (S: stem, M: multiloop, I: internal loop, B: bulge, E: dangling end, X: external loop) (**D**) Bar plot of F1 scores of RSS predictions by different algorithms for four recently released RNA structures from the PDB (PDB IDs: 7KVT, 8I7N, 7U4A, and 7EAF).

To further characterize the generalizability of the model in unseen families, two particularly challenging sequences (URS0000D6ACEB_12908_1-61, URS0000D69420_12908_1-94) were selected for visualization with the Forna tool [37], which are from the bpRNA-new dataset. To determine the similarity between the predicted RNA SS and the ground truth, BEAGLE is implemented [38]. The visualization results show that, in both cases, TrioFold-lite markedly generates SS more akin to the ground truth, with structure similarities of 96.721 and 87.234 (Figure 6A,B), structure alignment shows that our algorithm generates motifs more accurately (Figure 6C), compared with other algorithms. Although the prediction of the SS differs for each base learner, the ensemble model can obtain results with less generalization error by assigning different weights to different base learners, as evidenced by the fact that the SS predicted by the TrioFold-lite is closer to the real structure in both cases.

Additionally, we found four experimentally determined RNA structures newly released after 1 June 2021 in the PDB database and ensured the exclusion of any sequences from these RNAs in the datasets employed for this study. The performance of ensemble models was compared with other methods (Figure 6D). We observed that in certain sequences, the performance of all algorithms was suboptimal. Take 7U4A (crystal structure of Zika virus xrRNA1 mutant) for example; the F1 scores for the majority of algorithms hover around 0.35. This observation may be correlated with the complicated pseudoknots present in the structure. In summary, TrioFold outperformed other methods on three (7KVT, 7U4A, and 7EAF) out of the four RNA structures and was listed as the second-best method for the remaining one structure (8I7N).

### 2.7. A User-Friendly Webserver for RSS Prediction and Analysis

**Figure 7:**
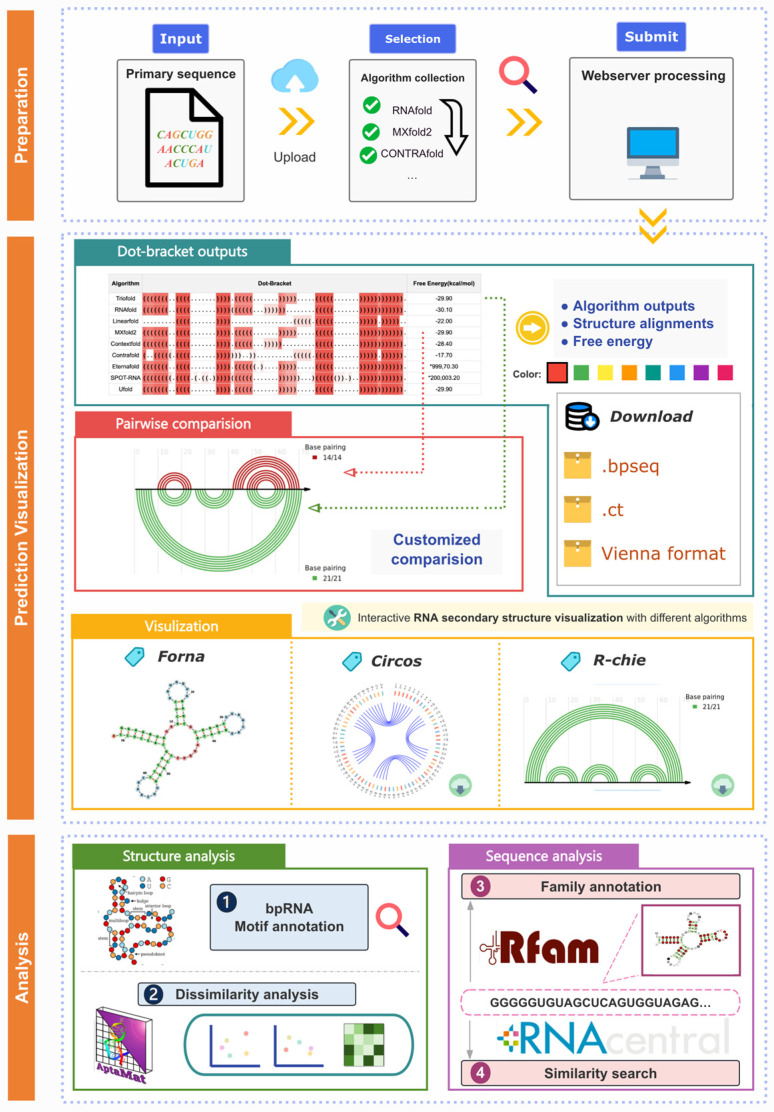
Workflow and functions of TrioFold webserver. The TrioFold webserver is a user-friendly platform that offers comprehensive RSS prediction and analysis. Additionally, the webserver provides custom RSS based on various methods, along with tools for motif annotation, dissimilarity analysis, and sequence analysis.

Moreover, we provide a user-friendly web interface integrated with RNA prediction and analysis (Figure 7). Users interested in utilizing the ensemble model can input the RNA primary sequence, and the site will invoke the TrioFold along with MXFold2, UFold, SPOT-RNA, EternaFold, CONTRAfold, RNAfold, LinearFold and ContextFold to generate RSS predictions. The output includes the predicted RSS from various algorithms and their alignments. The website also features a visualization window for intuitively observing and comparing the RSS predicted by various methods.

Based on this, the website also offers an analysis module based on RNA sequence and SS. The site integrates software including Forna (http://rna.tbi.univie.ac.at/forna/, accessed on 16 August 2025), Circos (https://circos.ca/, accessed on 16 August 2025) [39], e-RNA (https://www.e-rna.org/, accessed on 16 August 2025) [40], and Aptamat 1.0 [41]. Users can obtain information on RNA structure motifs and dissimilarity analysis is also available using different algorithms. Additionally, information on Rfam families and similar sequences from RNAcentral can also be obtained.

## 3. Materials and Methods

### 3.1. Sequence Module

The sequence module learns sequence information through various base learners B = (b_1_, ⋯, b*_l_*) to obtain the structure module’s input data, where l is the number of base learners. Given an RNA sequence x = (x_1_, ⋯, x*_n_*), where n is the sequence length, TrioFold inputs the sequence x into each base learner to generate an RSS described by dot–bracket notation.

Each base learner $b_{i}$ produces a binary matrix $A^{\left(i\right)}\in \;R^{n\;\times \;n}$ where:

- $A_{j,k}^{\left(i\right)}$ = 1 if the j-th base and the k-th base form a complementary pair according to the i-th base learner’s prediction.
- $A_{j,k}^{\left(i\right)}$ = 0 otherwise.

The complete output is represented as a 3-dimensional binary matrix:

(1) $$A=\left(A^{\left(1\right)},A^{\left(2\right)},\ldots ,A^{\left(l\right)}\right)\;\in R^{l\;\times n\;\times n}$$

### 3.2. Structure Module

To better integrate the strengths of base learners and compensate for their lack of generalization, we employ CBAM (Convolutional Block Attention Module) to capture important information from different learners and spatial locations. The overall ensemble process can be described as:

(2) $$A^{\prime }\;=\;F_{p}\left(A\right)\;⊗A$$

(3) $$A^{\prime \prime }=\;F_{d}\left(A^{\prime }\right)\;⊗A^{\prime }$$

where 1D RSS Pattern map $F_{P}$ ∈ $R^{l\;\times \;1\;\times \;1}$ and 2D Domain Focus map $F_{d}$ as illustrated in Figure 1, ⊗ denotes element-wise multiplication. $A^{\prime \prime }$ is the final refined output.

### 3.3. RSS Pattern Block

In RNA molecules, bases form specific pairings through hydrogen bonds (such as A-U, G-C, and G-U pairings), determining the RSS. We utilize the channel attention module from CBAM as the RSS Pattern Block to identify and reinforce important feature channels, thereby selecting and stabilizing key base-pair relationships. The RSS Pattern Block can be defined as follows:

(4) $$F_{P}\left(A\right)\;=\;\sigma \;\left(MLP\left(AvgPool\left(A\right)\right)+MLP\left(MaxPool\left(A\right)\right)\right)$$

where *σ* denotes the sigmoid function, and the *MLP* weights are shared for both inputs. *AvgPool* and *MaxPool* are applied along the spatial dimensions of the feature map while preserving the channel dimension, effectively aggregating information across the spatial locations.

### 3.4. Domain Focus Block

Key regions in RNA secondary structures, such as hairpin loops and internal loops, are crucial for RNA’s function and stability. We employ the spatial attention module from CBAM to enhance the feature representations of these critical regions, enabling the model to understand and predict RNA secondary structures better. The Domain Focus Block can be defined as follows:

(5) $$F_{d}\left(A^{\prime }\right)\;=\;\sigma \;(Conv([AvgPool\left(A^{\prime }\right),MaxPool(A^{\prime })]))$$

where σ denotes the sigmoid function, and Conv represents a convolution operation with the filter size of 7 × 7. This block applies AvgPool and MaxPool along the channel dimension, aggregating spatial information across all channels to generate two spatial context descriptors.

### 3.5. TrioFold-Lite

For TrioFold-lite, we employ a learnable ensemble network [42] that is capable of assigning weights to the matrices produced by different base learners. Firstly, we obtain the weighted sum of adjacent matrices through the following formula:

(6) $$E_{s}=\sum_{v=1}^{V}\left(\pi^{\left(v\right)}E^{\left(v\right)}\right)$$

where $E^{\left(v\right)}$ is the adjacent matrix output by *v*-th base learner, and $\pi^{\left(v\right)}$ is the automatically learned weight parameter optimized using gradient descent. To constrain the values of the adjacent matrix within the range of 0 and 1, we incorporate the softmax function into the network:

(7) $$\pi^{\left(v\right)}=\frac{exp(\pi^{\left(v\right)})}{\sum_{v=1}^{V}\pi^{\left(v\right)}}$$

### 3.6. Dataset

The datasets used in this study have been uniformly processed in the following steps: (1) CD-HIT-EST [43] is used to remove sequences with similarity > 80% to enhance the sampling representativeness of training data, to ensure the ability of the model to learn important features; (2) screen and filter some invalid sequences (such as some sequences with missing nucleotides); (3) remove RNA sequences containing more than 500 nucleotides in the database (except TestSetA) due to the limitation of computer resources. The datasets used in this study are as follows:

The Archive II dataset is a benchmark dataset established by Mathews [44]. The dataset contains 3975 RNA sequences from 10 RNA families, including small subunit ribosomal RNA, large subunit ribosomal RNA, 5S ribosomal RNA, Group I self-splicing introns, RNase P RNA, signal recognition particle RNA, tRNA, and tmRNA. All RSS in this dataset were obtained by comparative sequence analysis. This benchmark dataset is one of the most widely used RSS test sets.

The TS0 dataset is derived from the benchmark dataset established by Jaswinder [25]. The main source of this benchmark dataset is the bpRNA-1m 1.0 database [45]. The bpRNA database contains a wide range of RNA families. This dataset contains more than 100,000 sequences from 2588 RNA families. Therefore, it is suitable for unseen RNA family evaluation experiments of ensemble models. In this study, we use the same train-test split method as UFold. In detail, the bpRNA is divided into three datasets: TR0, VL0, and TS0, and only TS0 is used for experimental evaluation.

The bpRNA-new dataset is derived from the benchmark dataset established by Kengo [24]. The main source of the benchmark dataset is the Rfam14.2 database [46]. This benchmark dataset provides rich RNA family information and helps to verify the generalization ability of the model in different RNA families.

The PDB dataset is derived from the benchmark dataset established by Jaswinder [35], which can be further divided into TS1, TS2, and TS3. The main source of this benchmark dataset is the high-resolution RNA structure released by the PDB database [47]. In the PDB dataset, according to the method of SPOT-RNA2, homologous sequences were removed using BLAST-N software (https://blast.ncbi.nlm.nih.gov/Blast.cgi, accessed on 16 August 2025) [48] with an e-value cutoff of 10. These structures are non-redundant with the existing training, validation, and test sets.

TestSetA and TestSetB datasets are derived from the benchmark dataset established by Rivas [49]. The main source of the benchmark dataset is the Rfam 10.0 database. Test Seta keeps sequences greater than 500 nt, with sequence lengths ranging from 10 to 768 nt. It mainly contains 31 RNA families, such as tRNA, SRP RNA, 5sRNA, tmRNA, telomerase RNA, etc. The sequence length in TestSetB varies from 27 to 244 nt. The RNA family contained in TestSetA is completely different from the RNA family contained in TestSetB, so it can be considered that TestSetA and TestSetB are independent of each other. For TestSetA and TestSetB, after the above pre-processing steps, the method used by MXfold2 is used to remove the sequences that contain pseudoknot structures in the dataset.

For the TrioFold, to avoid overfitting, TestSetB and ArchiveII are used as the training set, and the sequences contained in the test set are removed. We used TestSetA as the validation set and evaluated model performance further on the bpRNA-new, TS0, TS-inter and PDB datasets. To assess sequence similarity between the training and evaluation datasets, we performed sequence identity analysis using VSEARCH [36]. We then trained this model for 100 epochs, saved the model at epoch 15 according to accuracy on the TestSetA, and used this model for subsequent ensemble model performance valuation.

### 3.7. Loss Function

RSS prediction is a typical binary classification problem. Binary cross entropy (BCE) loss function is a loss function invented for binary classification problems. The formula is as follows:

(8) $$BCE=\;\;-\frac{1}{N}\sum_{i=1}^{N}\left(y_{i}\log(\sigma (p_{i}))\;+\;{(1-\;y}_{i})\log\left(1-\;{\sigma (p}_{i})\right)\right)$$

where *N* represents the number of samples, $y_{i}$ represents the real label, and $p_{i}$ represents the base-pairing probability output by the model.

### 3.8. Baselines

In our study, we classify and introduce several baseline models to provide a comprehensive comparison with TrioFold. These baselines are grouped based on their underlying methodologies:

- Machine Learning-Based Models: Algorithms such as UFold (available at https://github.com/uci-cbcl/UFold, accessed on 16 August 2025), CONTRAfold, ContextFold, MXfold2 (available at https://github.com/mxfold/mxfold2, accessed on 16 August 2025), EternaFold (available at https://github.com/eternagame/EternaFold, accessed on 16 August 2025) and SPOT-RNA (available at https://github.com/jaswindersingh2/SPOT-RNA, accessed on 16 August 2025) leverage machine learning techniques, particularly deep learning and probabilistic models, to predict RNA secondary structures. These models are trained on large RNA datasets to capture complex sequence-structure relationships, offering high accuracy for various RNA types.
- Thermodynamic and Energy-Based Models: RNAfold (available at https://www.tbi.univie.ac.at/RNA/, accessed on 16 August 2025), Mfold (available at http://www.unafold.org/mfold/software/download-mfold.php, accessed on 16 August 2025), and LinearFold (available at https://linearfold.eecs.oregonstate.edu/, accessed on 16 August 2025) rely on thermodynamic principles, where RNA secondary structures are predicted by minimizing the free energy of the sequence. LinearFold, while not a machine learning method itself, integrates parameters from CONTRAfold, a machine learning-based model, to enhance its prediction efficiency without requiring a machine learning framework for its operation. These classical approaches are widely recognized for their robustness in RNA structure prediction based on energy calculations.

To ensure fairness and accuracy in our comparisons, the implementation and default parameter settings for each model strictly follow the original papers.

### 3.9. Implementation Details

In this study, the data pre-processing and training model are mainly completed on a server equipped with four NVIDIA GeForce RTX 3090. The CPU used is an Intel (R) Xeon (R) Gold 6230R CPU @ 2.10GHz, Python version is 3.9.16, and Pytorch version [50] is 1.10.0. The Linux system version number is Ubuntu 20.04.4 LTS, the optimizer selected for training is Adam optimizer, and its default parameters are set as training parameters. The batch size is set to 4. Due to the unbalanced proportion of positive and negative samples, the positive class weight is set to 300 to balance the weight of positive and negative samples during training.

### 3.10. Post-Process

It is widely accepted that the RSS should be restricted by certain hard constraints [51]. For a given sequence x = (x_1_, ⋯, x_n_), with a length of n, the contact map of RSS can be defined as a binary matrix Ζ*. If the i-th base in the sequence is complementary to the *j*-th base, then ${Ζ}_{i,j}\;$= 1, otherwise ${Ζ}_{i,j}\;$= 0. To make the output of the ensemble model more consistent with the actual RSS, the output structure should follow the following rules: (1) in the predicted structure, only canonical base pairings plus G-U pairing are allowed; (2) the contact map of the model output must be symmetrical; (3) paired bases should be at least four bases apart, where ∀*i*, *j*, |*i* − *j*| < 4, ${\;Ζ}_{i,j}$ = 0; (4) a base can only be paired with only one other base, regardless of a base pairing with two bases at the same time. That is, ∀*i*, $\sum_{j=1}^{n}\;{Ζ}_{i,j}$ ≤ 1.

To meet the above constraints, we use the post-processing method used by E2Efold [52]. The output matrix of the model without post-processing is defined as Y. Matrix M is defined as: ${M\left(x\right)}_{i,j}$ = 1 if $x_{i}x_{i}$ meets constraints 1 and 2, and ${M\left(x\right)}_{i,j}$ = 0 otherwise. By defining the following nonlinear transformation ϕ(Y), the matrix satisfying constraints 1, 2 and 3 can be obtained:

(9) $$\phi \left(Y\right)=\;\frac{1}{2}\left(Y+\;Y^{T}\right)\;*\;M\left(x\right)$$

To satisfy constraint 4, the problem can be transformed into a linear programming problem, where $\hat{Y}$ is the output of the previous iteration, and ρ is a hyperparameter that controls the sparsity of the output matrix. Through the optimization of the output matrix by this algorithm, a matrix with high similarity to $\phi \left(Y\right)$ while satisfying constraint 4 can be obtained.

(10) $${\hat{Y}}^{*}=\mathrm{argmax}\;\langle \hat{Y},\;\phi \left(Y\right)\rangle -\rho \left\|\hat{Y}\right\|,\;s.t.\;\hat{Y}\;\leq \;1$$

Finally, we set the threshold P. Let the element in the output that is greater than the threshold P be set to 1, otherwise 0, and the final binary matrix can be obtained.

### 3.11. Experimental Evaluation of TrioFold

Since the RSS prediction is to predict whether the bases of the corresponding sites can be paired, the problem can be defined as a binary classification problem, and the confusion matrix is used to classify the predicted results.

The commonly used metrics for RSS are recall, precision, F1 score, etc. Instead of using MCC, we adopt interaction network fidelity (INF), which is numerically equivalent to MCC but more appropriately captures the base-pairing interactions characteristic of RNA structure prediction [53]. The metrics as shown in the formulas below:

(11) $$precision\;=\;\frac{TP}{TP+FP}$$

(12) $$recall=\frac{TP}{TP+FN}$$

(13) $$F1\;score=\frac{2\;*\;precision\;*\;recall}{precision+recall}$$

(14) $$INF=\frac{TP\;*\;TN-FP\;*\;FN}{\sqrt{\left(TP+FP)(TN+FP)(TP+FN)(TN+FN\right)}}$$

If the predicted base pairs also exist in the actual structure, such cases are called true positive (*TP*); if the predicted base pair does not exist in the actual structure, such cases are called false positive (*FP*); if it is predicted that a base is not paired, and the base pair does not exist in the actual structure, such cases are defined as true negative (*TN*); if a predicted base pair does not exist in practice, such a case is defined as a false negative (*FN*).

To measure the prediction differences for different base learners, we use the Jaccard distance as a metric to determine dissimilarities. The calculation formula of the Jaccard distance is as follows.

(15) $$J\left(A,B\right)=\frac{\left|A⋃B\right|-\left|A⋂B\right|}{\left|A⋃B\right|}$$

where *A* and *B* are the contact maps for a single RNA sequence’s SS prediction computed by the base learners, respectively. The greater the Jaccard distance, the greater the difference between the prediction results of the two base learners.

### 3.12. Principal Component Analysis

For PCA, we reshaped the MSE of each structure calculated according to the ground truth structure into a one-dimensional matrix and used the resulting matrix as features for PCA analysis.

### 3.13. Webserver

A webserver was developed based on HTML5, PHP, Apache, etc. to provide the predictions using TrioFold and other widely used algorithms including CONTRAfold, MXfold2, UFold, ContextFold, EternaFold, LinearFold, RNAfold, and SPOT-RNA. The webserver can be freely visited at http://triofold.aiddlab.com/ (accessed on 16 August 2025). The input sequence length is limited due to computational resource constraints.

## 4. Discussion and Conclusions

Augmented by DL algorithms, RSS prediction algorithms have made huge leaps over the past several years. However, the generalizability of existing algorithms is usually unsatisfactory, which is inevitable to some extent because of the data-dependency nature of machine learning algorithms [54]. Due to the small amount and low quality of existing RNA structure data, the highly imbalanced distribution of RNA families, and the limitations in RNA sample representation strategies, DL-based RSS prediction algorithms often suffer from overfitting problems. The huge number of model parameters may lead to overfitting and poor generalizability [55]. To circumvent potential overfitting, we develop our method based on ensemble modules, thereby succeeding in mitigating the pitfalls associated with overparameterization. We hope this study will contribute ideas that may prove valuable in enhancing the generalizability and performance of DL-based RSS prediction algorithms.

Collectively, in this study, we present an end-to-end algorithm TrioFold and TrioFold-lite, based on ensemble learning and convolutional attention mechanisms to predict RSS. Our method successfully improves the accuracy of existing RSS prediction algorithms and alleviates the potential overfitting problem commonly occurring in ML/DL-based models, offering insights into strategies for enhancing both generalizability and overall performance. Although we did not retrain base learners, and they may have overfitting problems in predicting certain types of RNA, our algorithm still outperformed them. Furthermore, we have established a webserver to provide users with RSS prediction and analysis functions. By submitting the RNA sequences, users can acquire comprehensive information, including prediction results from various algorithms, structure visualization, and key properties (e.g., minimal free energy) of individual RSS automatically, all within a one-stop platform workflow, thereby enabling a more extensive utilization by biologists in a programming-free manner.

### Limitations of the Study

Although we demonstrated that the TrioFold/TrioFold-lite exhibits enhanced generalizability and accuracy in predicting both intra-family and inter-family sequences, there are still limitations in our study. The input of this model is solely the primary sequence of RNA; the integration of other information can be further considered, such as experiment-derived SHAPE reactivity [56] and multiple sequence alignment (MSA)-derived features, to further enhance the accuracy of RSS prediction. In addition, it is important to note the existence of numerous other algorithms in the field of RSS prediction. By expanding the scope to incorporate additional algorithms as the choice of base learners, especially those able to predict RNA structures with pseudoknots [57], the performance of TrioFold/TrioFold-lite may be further improved.

## Figures and Tables

**Table 1:** Performance on the bpRNA-new dataset. TrioFold/TrioFold-lite’s margin of F1 score over the remaining algorithms is listed. The highest values for each metric are highlighted in bold. **: *p* < 0.01.

Algorithm	INF	Precision	Recall	F1 Score	Margin of TrioFold	Margin of TrioFold-Lite
UFold	0.617	0.537	0.723	0.608	8.39% (**)	7.73% (**)
SPOT-RNA	0.608	0.593	0.641	0.603	9.29% (**)	8.62% (**)
ContextFold	0.584	0.546	0.636	0.580	13.62% (**)	12.93% (**)
CONTRAfold	0.646	0.579	**0.737**	0.639	3.13% (**)	2.50% (**)
Mfold	0.608	0.541	0.696	0.601	9.65% (**)	8.99% (**)
RNAfold	0.624	0.552	0.720	0.617	6.81% (**)	6.16% (**)
LinearFold	0.623	0.649	0.645	0.617	6.81% (**)	6.16% (**)
MXfold2	0.639	0.580	0.718	0.633	4.11% (**)	3.48% (**)
EternaFold	0.641	0.569	**0.737**	0.633	4.11% (**)	3.48% (**)
TrioFold	**0.665**	0.614	0.736	**0.659**	/	−0.61% (**)
TrioFold-lite	0.661	**0.627**	0.717	0.655	0.61% (**)	/

**Table 2:** Performance on the PDB dataset. TrioFold/TrioFold-lite’s margin of F1 score over the remining algorithms is listed. The highest values for each metric are highlighted in bold. n.s.: not significant (*p* ≥ 0.05); *: *p* < 0.05; **: *p* < 0.01.

Algorithm	INF	Precision	Recall	F1 Score	Margin of TrioFold	Margin of TrioFold-Lite
UFold	0.692	0.728	0.670	0.693	7.50% (**)	7.07% (**)
SPOT-RNA	0.751	0.852	**0.675**	0.744	0.13% (n.s.)	−0.27% (n.s.)
ContextFold	0.677	0.796	0.587	0.669	11.36% (**)	10.91% (**)
CONTRAfold	0.688	0.780	0.618	0.684	8.92% (**)	8.48% (**)
Mfold	0.668	0.763	0.596	0.664	12.20% (**)	11.75% (**)
RNAfold	0.683	0.776	0.612	0.680	9.56% (**)	9.12% (**)
LinearFold	0.660	0.803	0.568	0.651	14.44% (**)	13.98% (**)
MXfold2	0.707	0.829	0.620	0.700	6.43% (**)	6.00% (*)
EternaFold	0.687	0.776	0.619	0.684	8.92% (**)	8.48% (**)
TrioFold	0.752	0.865	0.666	**0.745**	/	−0.40% (n.s.)
TrioFold-lite	**0.754**	**0.888**	0.654	0.742	0.40% (n.s.)	/

**Table 3:** Performance on the TS-inter dataset. TrioFold/TrioFold-lite’s margin of F1 score over the remaining algorithms is listed. The highest values for each metric are highlighted in bold. n.s.: not significant (*p* ≥ 0.05); *: *p* < 0.05; **: *p* < 0.01.

Algorithm	INF	Precision	Recall	F1 Score	Margin of TrioFold	Margin of TrioFold-Lite
UFold	0.642	0.585	0.720	0.634	0.32% (n.s.)	2.68% (n.s.)
SPOT-RNA	0.602	0.550	0.678	0.594	7.07% (**)	9.60% (**)
ContextFold	0.546	0.488	0.634	0.536	18.66% (**)	21.46% (**)
CONTRAfold	0.543	0.475	0.640	0.532	19.55% (**)	22.37% (**)
Mfold	0.517	0.445	0.625	0.505	25.94% (**)	28.91% (**)
RNAfold	0.488	0.413	0.602	0.475	33.89% (**)	37.05% (**)
LinearFold	0.510	0.572	0.532	0.502	26.69% (**)	29.68% (**)
MXfold2	0.570	0.501	0.678	0.557	14.18% (**)	16.88% (**)
EternaFold	0.530	0.459	0.638	0.517	23.02% (**)	25.92% (**)
TrioFold	0.645	0.594	**0.722**	0.636	/	2.36% (*)
TrioFold-lite	**0.659**	**0.624**	0.713	**0.651**	−2.30% (*)	/

## Data Availability

The datasets used in this study have been deposited at https://doi.org/10.5281/zenodo.12714014 (accessed on 16 August 2025). The code is available at https://github.com/sfsdfd62/TrioFold (accessed on 16 August 2025) and https://figshare.com/articles/software/TrioFold/26377156 (accessed on 16 August 2025). The Supplementary Table S1 is also available at https://doi.org/10.5281/zenodo.15765109 (accessed on 16 August 2025).

## Supplementary Materials

The following supporting information can be downloaded at https://www.mdpi.com/article/10.3390/molecules30163447/s1.
