# Supplementary material for: Enhanced Generalizability of RNA Secondary Structure Prediction via Convolutional Block Attention Network and Ensemble Learning
Source: Molecules. 2025 Aug 21;30(16):3447. doi: 10.3390/molecules30163447 (PMC12388828; doi:10.3390/molecules30163447)
Supplement: Supplementary file 1 [file molecules-30-03447-s001.zip › Supplementary Table S1.pdf]

Supplementary Table S1: Performance of ensemble models with different base learner combinations

Combinations	F1 score	Precision	Recall
LinearFold, RNAfold, MXFold2, ContextFold, CONTRAfold, mfold	0.7329576	0.76139235	0.7419397
LinearFold, RNAfold, MXFold2, ContextFold, CONTRAfold, EternaFold	0.72797143	0.7581633	0.74066657
LinearFold, RNAfold, MXFold2, ContextFold, CONTRAfold, SPOT-RNA	0.76956594	0.79501957	0.7740582
LinearFold, RNAfold, MXFold2, ContextFold, CONTRAfold, UFold	0.7532056	0.7980941	0.75926423
LinearFold, RNAfold, MXFold2, ContextFold, mfold, EternaFold	0.73450166	0.7617392	0.744949
LinearFold, RNAfold, MXFold2, ContextFold, mfold, SPOT-RNA	0.7638665	0.7898854	0.7667387
LinearFold, RNAfold, MXFold2, ContextFold, mfold, UFold	0.7549377	0.79253113	0.76083785
LinearFold, RNAfold, MXFold2, ContextFold, EternaFold, SPOT-RNA	0.7707096	0.79481673	0.7757664
LinearFold, RNAfold, MXFold2, ContextFold, EternaFold, UFold	0.7598974	0.7987741	0.76702607
LinearFold, RNAfold, MXFold2, ContextFold, SPOT-RNA, UFold	0.7967254	0.8307617	0.7943965
LinearFold, RNAfold, MXFold2, CONTRAfold, mfold, EternaFold	0.71245193	0.73308986	0.73013234
LinearFold, RNAfold, MXFold2, CONTRAfold, mfold, SPOT-RNA	0.7318966	0.7593977	0.74066186
LinearFold, RNAfold, MXFold2, CONTRAfold, mfold, UFold	0.72156477	0.7605108	0.73142195
LinearFold, RNAfold, MXFold2, CONTRAfold, EternaFold, SPOT-RNA	0.7299042	0.75808835	0.74289477
LinearFold, RNAfold, MXFold2, CONTRAfold, EternaFold, UFold	0.725125	0.7593649	0.7391561
LinearFold, RNAfold, MXFold2, CONTRAfold, SPOT-RNA, UFold	0.7559437	0.79743403	0.7633452
LinearFold, RNAfold, MXFold2, mfold, EternaFold, SPOT-RNA	0.7353893	0.7616322	0.7462254
LinearFold, RNAfold, MXFold2, mfold, EternaFold, UFold	0.72505677	0.76373255	0.7352564
LinearFold, RNAfold, MXFold2, mfold, SPOT-RNA, UFold	0.7553498	0.78640664	0.76301724
LinearFold, RNAfold, MXFold2, EternaFold, SPOT-RNA, UFold	0.7589507	0.79784733	0.76734674
LinearFold, RNAfold, ContextFold, CONTRAfold, mfold, EternaFold	0.71281886	0.7405881	0.72376335
LinearFold, RNAfold, ContextFold, CONTRAfold, mfold, SPOT-RNA	0.7360773	0.76723367	0.7394704
LinearFold, RNAfold, ContextFold, CONTRAfold, mfold, UFold	0.7237466	0.771895	0.7281095
LinearFold, RNAfold, ContextFold, CONTRAfold, EternaFold, SPOT-RNA	0.72888726	0.7629647	0.7375689
LinearFold, RNAfold, ContextFold, CONTRAfold, EternaFold, UFold	0.7237549	0.76451904	0.7325361
LinearFold, RNAfold, ContextFold, CONTRAfold, SPOT-RNA, UFold	0.7600687	0.8063777	0.76334494
LinearFold, RNAfold, ContextFold, mfold, EternaFold, SPOT-RNA	0.737314	0.76733124	0.7423908
LinearFold, RNAfold, ContextFold, mfold, EternaFold, UFold	0.72808456	0.7719873	0.7330421

LinearFold, RNAfold, ContextFold, mfold, SPOT-RNA, UFold	0.76480335	0.80526435	0.76385784
LinearFold, RNAfold, ContextFold, EternaFold, SPOT-RNA, UFold	0.76553714	0.8073848	0.76836187
LinearFold, RNAfold, CONTRAfold, mfold, EternaFold, SPOT-RNA	0.7151835	0.7429363	0.72692496
LinearFold, RNAfold, CONTRAfold, mfold, EternaFold, UFold	0.7067381	0.7440932	0.7193404
LinearFold, RNAfold, CONTRAfold, mfold, SPOT-RNA, UFold	0.72479635	0.76885307	0.72887444
LinearFold, RNAfold, CONTRAfold, EternaFold, SPOT-RNA, UFold	0.72594655	0.7647293	0.7367926
LinearFold, RNAfold, mfold, EternaFold, SPOT-RNA, UFold	0.7273317	0.7706067	0.7320474
LinearFold, MXFold2, ContextFold, CONTRAfold, mfold, EternaFold	0.7375292	0.7636103	0.7480014
LinearFold, MXFold2, ContextFold, CONTRAfold, mfold, SPOT-RNA	0.77148646	0.7950783	0.7741153
LinearFold, MXFold2, ContextFold, CONTRAfold, mfold, UFold	0.7642162	0.79836273	0.7682675
LinearFold, MXFold2, ContextFold, CONTRAfold, EternaFold, SPOT-RNA	0.76603013	0.7896164	0.7733031
LinearFold, MXFold2, ContextFold, CONTRAfold, EternaFold, UFold	0.7550735	0.7915151	0.76591104
LinearFold, MXFold2, ContextFold, CONTRAfold, SPOT-RNA, UFold	0.7971355	0.83065075	0.79550457
LinearFold, MXFold2, ContextFold, mfold, EternaFold, SPOT-RNA	0.7730513	0.7985371	0.7745186
LinearFold, MXFold2, ContextFold, mfold, EternaFold, UFold	0.7667659	0.80182856	0.7714941
LinearFold, MXFold2, ContextFold, mfold, SPOT-RNA, UFold	0.79677314	0.83075476	0.792522
LinearFold, MXFold2, ContextFold, EternaFold, SPOT-RNA, UFold	0.79855096	0.8320422	0.79807186
LinearFold, MXFold2, CONTRAfold, mfold, EternaFold, SPOT-RNA	0.7378847	0.7611497	0.7488463
LinearFold, MXFold2, CONTRAfold, mfold, EternaFold, UFold	0.7323324	0.76219994	0.74473304
LinearFold, MXFold2, CONTRAfold, mfold, SPOT-RNA, UFold	0.7655566	0.79639643	0.77122605
LinearFold, MXFold2, CONTRAfold, EternaFold, SPOT-RNA, UFold	0.7579796	0.7896883	0.77025706
LinearFold, MXFold2, mfold, EternaFold, SPOT-RNA, UFold	0.7671337	0.7981194	0.77342516
LinearFold, ContextFold, CONTRAfold, mfold, EternaFold, SPOT-RNA	0.7384638	0.7666708	0.7443992
LinearFold, ContextFold, CONTRAfold, mfold, EternaFold, UFold	0.7294915	0.7678445	0.7362308
LinearFold, ContextFold, CONTRAfold, mfold, SPOT-RNA, UFold	0.76991904	0.8080055	0.7695917
LinearFold, ContextFold, CONTRAfold, EternaFold, SPOT-RNA, UFold	0.75964785	0.8016283	0.7670974
LinearFold, ContextFold, mfold, EternaFold, SPOT-RNA, UFold	0.77152383	0.81106645	0.7715471
LinearFold, CONTRAfold, mfold, EternaFold, SPOT-RNA, UFold	0.7329559	0.76541585	0.7416319
RNAfold, MXFold2, ContextFold, CONTRAfold, mfold, EternaFold	0.7383168	0.74899334	0.7582951
RNAfold, MXFold2, ContextFold, CONTRAfold, mfold, SPOT-RNA	0.7653971	0.77521485	0.77870077

RNAfold, MXFold2, ContextFold, CONTRAfold, mfold, UFold	0.76080024	0.77993405	0.77589685
RNAfold, MXFold2, ContextFold, CONTRAfold, EternaFold, SPOT-RNA	0.7669402	0.77764416	0.78389364
RNAfold, MXFold2, ContextFold, CONTRAfold, EternaFold, UFold	0.75744087	0.77838475	0.77636063
RNAfold, MXFold2, ContextFold, CONTRAfold, SPOT-RNA, UFold	0.7980116	0.81860393	0.8045475
RNAfold, MXFold2, ContextFold, mfold, EternaFold, SPOT-RNA	0.76522106	0.77467525	0.77953225
RNAfold, MXFold2, ContextFold, mfold, EternaFold, UFold	0.7615263	0.77968967	0.7780631
RNAfold, MXFold2, ContextFold, mfold, SPOT-RNA, UFold	0.7914415	0.8092954	0.7984456
RNAfold, MXFold2, ContextFold, EternaFold, SPOT-RNA, UFold	0.7971958	0.8151417	0.80558497
RNAfold, MXFold2, CONTRAfold, mfold, EternaFold, SPOT-RNA	0.737498	0.7502271	0.7562735
RNAfold, MXFold2, CONTRAfold, mfold, EternaFold, UFold	0.733534	0.7520141	0.75328165
RNAfold, MXFold2, CONTRAfold, mfold, SPOT-RNA, UFold	0.76063085	0.7774772	0.77788544
RNAfold, MXFold2, CONTRAfold, EternaFold, SPOT-RNA, UFold	0.7577768	0.7789608	0.77642024
RNAfold, MXFold2, mfold, EternaFold, SPOT-RNA, UFold	0.7620018	0.7792005	0.779788
RNAfold, ContextFold, CONTRAfold, mfold, EternaFold, SPOT-RNA	0.74188536	0.7565713	0.7563817
RNAfold, ContextFold, CONTRAfold, mfold, EternaFold, UFold	0.7361085	0.760034	0.74947035
RNAfold, ContextFold, CONTRAfold, mfold, SPOT-RNA, UFold	0.76773673	0.78794855	0.77925354
RNAfold, ContextFold, CONTRAfold, EternaFold, SPOT-RNA, UFold	0.7616052	0.78434944	0.7788008
RNAfold, ContextFold, mfold, EternaFold, SPOT-RNA, UFold	0.76677305	0.7880646	0.7782187
RNAfold, CONTRAfold, mfold, EternaFold, SPOT-RNA, UFold	0.73483616	0.7594987	0.7472516
MXFold2, ContextFold, CONTRAfold, mfold, EternaFold, SPOT-RNA	0.7704382	0.77994275	0.78336203
MXFold2, ContextFold, CONTRAfold, mfold, EternaFold, UFold	0.7661693	0.7826322	0.78283674
MXFold2, ContextFold, CONTRAfold, mfold, SPOT-RNA, UFold	0.79914236	0.81711644	0.80461836
MXFold2, ContextFold, CONTRAfold, EternaFold, SPOT-RNA, UFold	0.7963166	0.81431943	0.80574054
MXFold2, ContextFold, mfold, EternaFold, SPOT-RNA, UFold	0.7984903	0.8177941	0.8044019
MXFold2, CONTRAfold, mfold, EternaFold, SPOT-RNA, UFold	0.76507866	0.7809591	0.78178436
ContextFold, CONTRAfold, mfold, EternaFold, SPOT-RNA, UFold	0.7702562	0.78769565	0.78396684
LinearFold, RNAfold, MXFold2, ContextFold, CONTRAfold, mfold, EternaFold	0.7197071	0.78148615	0.71047425
LinearFold, RNAfold, MXFold2, ContextFold, CONTRAfold, mfold, SPOT-RNA	0.7396146	0.80882275	0.7217968
LinearFold, RNAfold, MXFold2, ContextFold, CONTRAfold, mfold, UFold	0.7271813	0.80833066	0.7108593
LinearFold, RNAfold, MXFold2, ContextFold, CONTRAfold, EternaFold, SPOT-RNA	0.7344989	0.8022602	0.7219005

LinearFold, RNAfold, MXFold2, ContextFold, CONTRAfold, EternaFold, UFold	0.7303603	0.8053767	0.7176588
LinearFold, RNAfold, MXFold2, ContextFold, CONTRAfold, SPOT-RNA, UFold	0.7576031	0.8379751	0.73778343
LinearFold, RNAfold, MXFold2, ContextFold, mfold, EternaFold, SPOT-RNA	0.7419848	0.81039995	0.7257173
LinearFold, RNAfold, MXFold2, ContextFold, mfold, EternaFold, UFold	0.7320042	0.8085538	0.7161185
LinearFold, RNAfold, MXFold2, ContextFold, mfold, SPOT-RNA, UFold	0.75918895	0.83639145	0.73708713
LinearFold, RNAfold, MXFold2, ContextFold, EternaFold, SPOT-RNA, UFold	0.7622936	0.8385136	0.74301314
LinearFold, RNAfold, MXFold2, CONTRAfold, mfold, EternaFold, SPOT-RNA	0.7215709	0.77910227	0.7128173
LinearFold, RNAfold, MXFold2, CONTRAfold, mfold, EternaFold, UFold	0.71221364	0.78021324	0.7040979
LinearFold, RNAfold, MXFold2, CONTRAfold, mfold, SPOT-RNA, UFold	0.7284089	0.80574244	0.71245384
LinearFold, RNAfold, MXFold2, CONTRAfold, EternaFold, SPOT-RNA, UFold	0.7324798	0.80254006	0.72137123
LinearFold, RNAfold, MXFold2, mfold, EternaFold, SPOT-RNA, UFold	0.7316429	0.80665153	0.71657777
LinearFold, RNAfold, ContextFold, CONTRAfold, mfold, EternaFold, SPOT-RNA	0.71745855	0.78421545	0.70447415
LinearFold, RNAfold, ContextFold, CONTRAfold, mfold, EternaFold, UFold	0.7105818	0.7853505	0.69924295
LinearFold, RNAfold, ContextFold, CONTRAfold, mfold, SPOT-RNA, UFold	0.72871023	0.8191721	0.70604867
LinearFold, RNAfold, ContextFold, CONTRAfold, EternaFold, SPOT-RNA, UFold	0.73185915	0.81237197	0.7139826
LinearFold, RNAfold, ContextFold, mfold, EternaFold, SPOT-RNA, UFold	0.73450667	0.8213866	0.71218127
LinearFold, RNAfold, CONTRAfold, mfold, EternaFold, SPOT-RNA, UFold	0.7122866	0.7817815	0.70166284
LinearFold, MXFold2, ContextFold, CONTRAfold, mfold, EternaFold, SPOT-RNA	0.743289	0.8051397	0.72897
LinearFold, MXFold2, ContextFold, CONTRAfold, mfold, EternaFold, UFold	0.73567706	0.8074895	0.721202
LinearFold, MXFold2, ContextFold, CONTRAfold, mfold, SPOT-RNA, UFold	0.76601285	0.83972883	0.7438688
LinearFold, MXFold2, ContextFold, CONTRAfold, EternaFold, SPOT-RNA, UFold	0.76186246	0.8340873	0.74449337
LinearFold, MXFold2, ContextFold, mfold, EternaFold, SPOT-RNA, UFold	0.7669145	0.83932436	0.7460828
LinearFold, MXFold2, CONTRAfold, mfold, EternaFold, SPOT-RNA, UFold	0.7393254	0.80677485	0.7260058
LinearFold, ContextFold, CONTRAfold, mfold, EternaFold, SPOT-RNA, UFold	0.7363953	0.8184398	0.7163038
RNAfold, MXFold2, ContextFold, CONTRAfold, mfold, EternaFold, SPOT-RNA	0.7454605	0.79022473	0.73946506
RNAfold, MXFold2, ContextFold, CONTRAfold, mfold, EternaFold, UFold	0.73911256	0.7929008	0.7331553
RNAfold, MXFold2, ContextFold, CONTRAfold, mfold, SPOT-RNA, UFold	0.76664305	0.82584274	0.7521647
RNAfold, MXFold2, ContextFold, CONTRAfold, EternaFold, SPOT-RNA, UFold	0.7656563	0.82503843	0.755598
RNAfold, MXFold2, ContextFold, mfold, EternaFold, SPOT-RNA, UFold	0.766308	0.8255453	0.75319755
RNAfold, MXFold2, CONTRAfold, mfold, EternaFold, SPOT-RNA, UFold	0.74007946	0.79104424	0.73448974

RNAfold, ContextFold, CONTRAfold, mfold, EternaFold, SPOT-RNA, UFold	0.7378085	0.79783005	0.7273978
MXFold2, ContextFold, CONTRAfold, mfold, EternaFold, SPOT-RNA, UFold	0.7714045	0.8252108	0.75913554
LinearFold, RNAfold, MXFold2, ContextFold, CONTRAfold, mfold, EternaFold, SPOT-RNA	0.74330974	0.7776696	0.7464703
LinearFold, RNAfold, MXFold2, ContextFold, CONTRAfold, mfold, EternaFold, UFold	0.7363132	0.7792635	0.7398379
LinearFold, RNAfold, MXFold2, ContextFold, CONTRAfold, mfold, SPOT-RNA, UFold	0.7637529	0.8067499	0.761752
LinearFold, RNAfold, MXFold2, ContextFold, CONTRAfold, EternaFold, SPOT-RNA, UFold	0.759886	0.80377406	0.7627511
LinearFold, RNAfold, MXFold2, ContextFold, mfold, EternaFold, SPOT-RNA, UFold	0.7638923	0.8081808	0.762681
LinearFold, RNAfold, MXFold2, CONTRAfold, mfold, EternaFold, SPOT-RNA, UFold	0.738394	0.77843255	0.7420612
LinearFold, RNAfold, ContextFold, CONTRAfold, mfold, EternaFold, SPOT-RNA, UFold	0.736139	0.782499	0.7369226
LinearFold, MXFold2, ContextFold, CONTRAfold, mfold, EternaFold, SPOT-RNA, UFold	0.766536	0.80453867	0.7670613
RNAfold, MXFold2, ContextFold, CONTRAfold, mfold, EternaFold, SPOT-RNA, UFold	0.765493	0.79387456	0.7736028
LinearFold, RNAfold, MXFold2, ContextFold, CONTRAfold, mfold, EternaFold, SPOT-RNA, UFold	0.7395524	0.812506	0.7240056
LinearFold, RNAfold, MXFold2, ContextFold, CONTRAfold	0.72276366	0.79808784	0.70885944
LinearFold, RNAfold, MXFold2, ContextFold, mfold	0.7233893	0.7947238	0.70748574
LinearFold, RNAfold, MXFold2, ContextFold, EternaFold	0.72889924	0.8005699	0.7155726
LinearFold, RNAfold, MXFold2, ContextFold, SPOT-RNA	0.770546	0.835259	0.74744886
LinearFold, RNAfold, MXFold2, ContextFold, UFold	0.7454931	0.84066606	0.7244336
LinearFold, RNAfold, MXFold2, CONTRAfold, mfold	0.69909525	0.7628016	0.6922868
LinearFold, RNAfold, MXFold2, CONTRAfold, EternaFold	0.69948894	0.75687355	0.69753283
LinearFold, RNAfold, MXFold2, CONTRAfold, SPOT-RNA	0.7236172	0.7944954	0.71077454
LinearFold, RNAfold, MXFold2, CONTRAfold, UFold	0.71487844	0.799595	0.702664
LinearFold, RNAfold, MXFold2, mfold, EternaFold	0.70174736	0.764409	0.69610363
LinearFold, RNAfold, MXFold2, mfold, SPOT-RNA	0.7220556	0.7876917	0.7072525
LinearFold, RNAfold, MXFold2, mfold, UFold	0.70393175	0.7913766	0.69134456
LinearFold, RNAfold, MXFold2, EternaFold, SPOT-RNA	0.72792155	0.79674506	0.7163212
LinearFold, RNAfold, MXFold2, EternaFold, UFold	0.71713585	0.7999748	0.70641464
LinearFold, RNAfold, MXFold2, SPOT-RNA, UFold	0.7470939	0.8387628	0.72904974
LinearFold, RNAfold, ContextFold, CONTRAfold, mfold	0.6996138	0.7768885	0.6860847
LinearFold, RNAfold, ContextFold, CONTRAfold, EternaFold	0.69931096	0.7634088	0.69285524
LinearFold, RNAfold, ContextFold, CONTRAfold, SPOT-RNA	0.7227829	0.8069355	0.70137566

LinearFold, RNAfold, ContextFold, CONTRAfold, UFold	0.7136731	0.81384176	0.6940342
LinearFold, RNAfold, ContextFold, mfold, EternaFold	0.7029368	0.7730157	0.6904583
LinearFold, RNAfold, ContextFold, mfold, SPOT-RNA	0.72698885	0.8099972	0.7001315
LinearFold, RNAfold, ContextFold, mfold, UFold	0.7079507	0.8155415	0.68337804
LinearFold, RNAfold, ContextFold, EternaFold, SPOT-RNA	0.7296139	0.80988425	0.70884395
LinearFold, RNAfold, ContextFold, EternaFold, UFold	0.71660924	0.8136572	0.69724053
LinearFold, RNAfold, ContextFold, SPOT-RNA, UFold	0.7520642	0.8575949	0.724169
LinearFold, RNAfold, CONTRAfold, mfold, EternaFold	0.67960584	0.74165016	0.67588496
LinearFold, RNAfold, CONTRAfold, mfold, SPOT-RNA	0.69894844	0.7691984	0.6852124
LinearFold, RNAfold, CONTRAfold, mfold, UFold	0.69003004	0.7760132	0.6781058
LinearFold, RNAfold, CONTRAfold, EternaFold, SPOT-RNA	0.69848835	0.7621455	0.6929592
LinearFold, RNAfold, CONTRAfold, EternaFold, UFold	0.69340205	0.763852	0.6887327
LinearFold, RNAfold, CONTRAfold, SPOT-RNA, UFold	0.71858186	0.80930644	0.70014524
LinearFold, RNAfold, mfold, EternaFold, SPOT-RNA	0.7005629	0.7719526	0.68797636
LinearFold, RNAfold, mfold, EternaFold, UFold	0.6910677	0.77862054	0.68097436
LinearFold, RNAfold, mfold, SPOT-RNA, UFold	0.70694727	0.81365764	0.6829467
LinearFold, RNAfold, EternaFold, SPOT-RNA, UFold	0.7190755	0.811711	0.70086926
LinearFold, MXFold2, ContextFold, CONTRAfold, mfold	0.73246914	0.7995637	0.7143148
LinearFold, MXFold2, ContextFold, CONTRAfold, EternaFold	0.7243684	0.788183	0.71661395
LinearFold, MXFold2, ContextFold, CONTRAfold, SPOT-RNA	0.7719802	0.83447254	0.75001556
LinearFold, MXFold2, ContextFold, CONTRAfold, UFold	0.75341964	0.83936083	0.7336222
LinearFold, MXFold2, ContextFold, mfold, EternaFold	0.73830783	0.80494475	0.72204256
LinearFold, MXFold2, ContextFold, mfold, SPOT-RNA	0.76988155	0.8377303	0.743497
LinearFold, MXFold2, ContextFold, mfold, UFold	0.756413	0.8417667	0.7321771
LinearFold, MXFold2, ContextFold, EternaFold, SPOT-RNA	0.7730489	0.83665633	0.75175273
LinearFold, MXFold2, ContextFold, EternaFold, UFold	0.75999534	0.84068656	0.73999864
LinearFold, MXFold2, ContextFold, SPOT-RNA, UFold	0.78967965	0.86600274	0.7635581
LinearFold, MXFold2, CONTRAfold, mfold, EternaFold	0.7060279	0.7603578	0.70095193
LinearFold, MXFold2, CONTRAfold, mfold, SPOT-RNA	0.7344526	0.7991745	0.71832836
LinearFold, MXFold2, CONTRAfold, mfold, UFold	0.7209795	0.80079395	0.70470023

LinearFold, MXFold2, CONTRAfold, EternaFold, SPOT-RNA	0.7254656	0.78470606	0.7173679
LinearFold, MXFold2, CONTRAfold, EternaFold, UFold	0.7217124	0.7905745	0.7132879
LinearFold, MXFold2, CONTRAfold, SPOT-RNA, UFold	0.758369	0.8376919	0.7403511
LinearFold, MXFold2, mfold, EternaFold, SPOT-RNA	0.73901737	0.8023577	0.7244137
LinearFold, MXFold2, mfold, EternaFold, UFold	0.72620296	0.80375445	0.71198726
LinearFold, MXFold2, mfold, SPOT-RNA, UFold	0.7591574	0.83905935	0.7378955
LinearFold, MXFold2, EternaFold, SPOT-RNA, UFold	0.76113284	0.83742607	0.7445735
LinearFold, ContextFold, CONTRAfold, mfold, EternaFold	0.70536363	0.7670948	0.69565386
LinearFold, ContextFold, CONTRAfold, mfold, SPOT-RNA	0.7336672	0.8118198	0.70852226
LinearFold, ContextFold, CONTRAfold, mfold, UFold	0.71942043	0.8145343	0.6953523
LinearFold, ContextFold, CONTRAfold, EternaFold, SPOT-RNA	0.72565335	0.79842734	0.7107072
LinearFold, ContextFold, CONTRAfold, EternaFold, UFold	0.7183665	0.803429	0.7031119
LinearFold, ContextFold, CONTRAfold, SPOT-RNA, UFold	0.75653315	0.85583234	0.72953254
LinearFold, ContextFold, mfold, EternaFold, SPOT-RNA	0.73701805	0.8147208	0.71332544
LinearFold, ContextFold, mfold, EternaFold, UFold	0.7245772	0.81688046	0.70104134
LinearFold, ContextFold, mfold, SPOT-RNA, UFold	0.7617647	0.8582525	0.73028576
LinearFold, ContextFold, EternaFold, SPOT-RNA, UFold	0.76147956	0.8554071	0.73396516
LinearFold, CONTRAfold, mfold, EternaFold, SPOT-RNA	0.7053542	0.7635334	0.6956764
LinearFold, CONTRAfold, mfold, EternaFold, UFold	0.69596463	0.7654973	0.68657297
LinearFold, CONTRAfold, mfold, SPOT-RNA, UFold	0.7235085	0.8136432	0.69993436
LinearFold, CONTRAfold, EternaFold, SPOT-RNA, UFold	0.7197689	0.80074877	0.7044929
LinearFold, mfold, EternaFold, SPOT-RNA, UFold	0.7285413	0.81914854	0.7065617
RNAfold, MXFold2, ContextFold, CONTRAfold, mfold	0.73384356	0.7763144	0.7291718
RNAfold, MXFold2, ContextFold, CONTRAfold, EternaFold	0.7328826	0.77528846	0.73323876
RNAfold, MXFold2, ContextFold, CONTRAfold, SPOT-RNA	0.7755921	0.8208397	0.7633
RNAfold, MXFold2, ContextFold, CONTRAfold, UFold	0.76141757	0.8258034	0.7504819
RNAfold, MXFold2, ContextFold, mfold, EternaFold	0.7354204	0.77598685	0.73272014
RNAfold, MXFold2, ContextFold, mfold, SPOT-RNA	0.7661197	0.81278867	0.751633
RNAfold, MXFold2, ContextFold, mfold, UFold	0.7564119	0.81679237	0.7452106
RNAfold, MXFold2, ContextFold, EternaFold, SPOT-RNA	0.7751675	0.81743705	0.76502764

RNAfold, MXFold2, ContextFold, EternaFold, UFold	0.7667304	0.8228365	0.7580158
RNAfold, MXFold2, ContextFold, SPOT-RNA, UFold	0.7990216	0.8516605	0.78208524
RNAfold, MXFold2, CONTRAfold, mfold, EternaFold	0.71482587	0.74821633	0.719232
RNAfold, MXFold2, CONTRAfold, mfold, SPOT-RNA	0.7314791	0.7735087	0.72691566
RNAfold, MXFold2, CONTRAfold, mfold, UFold	0.71960944	0.7748725	0.7159258
RNAfold, MXFold2, CONTRAfold, EternaFold, SPOT-RNA	0.73378867	0.77517533	0.7342001
RNAfold, MXFold2, CONTRAfold, EternaFold, UFold	0.7295785	0.77667344	0.7311066
RNAfold, MXFold2, CONTRAfold, SPOT-RNA, UFold	0.7619162	0.82261455	0.7533199
RNAfold, MXFold2, mfold, EternaFold, SPOT-RNA	0.7355887	0.7762095	0.73302203
RNAfold, MXFold2, mfold, EternaFold, UFold	0.723753	0.77825975	0.7207138
RNAfold, MXFold2, mfold, SPOT-RNA, UFold	0.7562116	0.8105857	0.74687177
RNAfold, MXFold2, EternaFold, SPOT-RNA, UFold	0.7627227	0.81968373	0.7559134
RNAfold, ContextFold, CONTRAfold, mfold, EternaFold	0.7153866	0.7562418	0.7132812
RNAfold, ContextFold, CONTRAfold, mfold, SPOT-RNA	0.73543215	0.7842693	0.7238912
RNAfold, ContextFold, CONTRAfold, mfold, UFold	0.7225742	0.7888215	0.712855
RNAfold, ContextFold, CONTRAfold, EternaFold, SPOT-RNA	0.73164093	0.7796752	0.72702336
RNAfold, ContextFold, CONTRAfold, EternaFold, UFold	0.72836226	0.7833739	0.72360045
RNAfold, ContextFold, CONTRAfold, SPOT-RNA, UFold	0.7700716	0.8423273	0.751603
RNAfold, ContextFold, mfold, EternaFold, SPOT-RNA	0.7375519	0.7845058	0.72778356
RNAfold, ContextFold, mfold, EternaFold, UFold	0.72663754	0.7891896	0.7171574
RNAfold, ContextFold, mfold, SPOT-RNA, UFold	0.76889724	0.8375402	0.747472
RNAfold, ContextFold, EternaFold, SPOT-RNA, UFold	0.77293795	0.84003645	0.7548268
RNAfold, CONTRAfold, mfold, EternaFold, SPOT-RNA	0.7170385	0.7587554	0.7157243
RNAfold, CONTRAfold, mfold, EternaFold, UFold	0.70796555	0.7611092	0.70702034
RNAfold, CONTRAfold, mfold, SPOT-RNA, UFold	0.72141063	0.78533536	0.7103728
RNAfold, CONTRAfold, EternaFold, SPOT-RNA, UFold	0.7270639	0.78217	0.72341067
RNAfold, mfold, EternaFold, SPOT-RNA, UFold	0.7240432	0.78648704	0.71405774
MXFold2, ContextFold, CONTRAfold, mfold, EternaFold	0.74235076	0.7810824	0.74011
MXFold2, ContextFold, CONTRAfold, mfold, SPOT-RNA	0.7766472	0.8201468	0.76217383
MXFold2, ContextFold, CONTRAfold, mfold, UFold	0.7711301	0.82397836	0.7581465

MXFold2, ContextFold, CONTRAfold, EternaFold, SPOT-RNA	0.7740399	0.8162898	0.76395047
MXFold2, ContextFold, CONTRAfold, EternaFold, UFold	0.7652948	0.819124	0.75872403
MXFold2, ContextFold, CONTRAfold, SPOT-RNA, UFold	0.80253696	0.8539635	0.7859907
MXFold2, ContextFold, mfold, EternaFold, SPOT-RNA	0.7762822	0.8204678	0.76246244
MXFold2, ContextFold, mfold, EternaFold, UFold	0.77235454	0.82494915	0.7610673
MXFold2, ContextFold, mfold, SPOT-RNA, UFold	0.7989887	0.85216445	0.7797783
MXFold2, ContextFold, EternaFold, SPOT-RNA, UFold	0.8008588	0.8530524	0.785726
MXFold2, CONTRAfold, mfold, EternaFold, SPOT-RNA	0.74177384	0.7787949	0.73954976
MXFold2, CONTRAfold, mfold, EternaFold, UFold	0.73706275	0.78067106	0.73618567
MXFold2, CONTRAfold, mfold, SPOT-RNA, UFold	0.77085805	0.82168347	0.7602335
MXFold2, CONTRAfold, EternaFold, SPOT-RNA, UFold	0.76561284	0.81625855	0.7603062
MXFold2, mfold, EternaFold, SPOT-RNA, UFold	0.7708258	0.8200323	0.7622931
ContextFold, CONTRAfold, mfold, EternaFold, SPOT-RNA	0.7412248	0.78557676	0.73237985
ContextFold, CONTRAfold, mfold, EternaFold, UFold	0.7343036	0.78802806	0.7265528
ContextFold, CONTRAfold, mfold, SPOT-RNA, UFold	0.7789545	0.8435099	0.75704134
ContextFold, CONTRAfold, EternaFold, SPOT-RNA, UFold	0.76927507	0.83576983	0.75421476
ContextFold, mfold, EternaFold, SPOT-RNA, UFold	0.77754885	0.842038	0.7575153
CONTRAfold, mfold, EternaFold, SPOT-RNA, UFold	0.73498726	0.784677	0.7284323
LinearFold, RNAfold, MXFold2, ContextFold	0.76362497	0.77278435	0.78313094
LinearFold, RNAfold, MXFold2, CONTRAfold	0.70886195	0.72638303	0.73612195
LinearFold, RNAfold, MXFold2, mfold	0.70475346	0.71979785	0.7292589
LinearFold, RNAfold, MXFold2, EternaFold	0.7147462	0.72467566	0.7443138
LinearFold, RNAfold, MXFold2, SPOT-RNA	0.76338303	0.7651691	0.7835819
LinearFold, RNAfold, MXFold2, UFold	0.73769015	0.7699083	0.76251954
LinearFold, RNAfold, ContextFold, CONTRAfold	0.71234494	0.7400194	0.7293313
LinearFold, RNAfold, ContextFold, mfold	0.7099397	0.72585416	0.73038596
LinearFold, RNAfold, ContextFold, EternaFold	0.7179801	0.7417832	0.7370096
LinearFold, RNAfold, ContextFold, SPOT-RNA	0.77109253	0.7915043	0.7795831
LinearFold, RNAfold, ContextFold, UFold	0.7493563	0.7939199	0.7631596
LinearFold, RNAfold, CONTRAfold, mfold	0.6839347	0.69914883	0.7125397

LinearFold, RNAfold, CONTRAfold, EternaFold	0.6881599	0.7083874	0.714673
LinearFold, RNAfold, CONTRAfold, SPOT-RNA	0.7160041	0.73707545	0.73607343
LinearFold, RNAfold, CONTRAfold, UFold	0.7056158	0.7424178	0.7261977
LinearFold, RNAfold, mfold, EternaFold	0.6845585	0.69865024	0.713356
LinearFold, RNAfold, mfold, SPOT-RNA	0.70658463	0.7212016	0.72585946
LinearFold, RNAfold, mfold, UFold	0.6895859	0.72554547	0.7114393
LinearFold, RNAfold, EternaFold, SPOT-RNA	0.7172025	0.7403405	0.7374287
LinearFold, RNAfold, EternaFold, UFold	0.7087472	0.74025077	0.7312752
LinearFold, RNAfold, SPOT-RNA, UFold	0.75310576	0.7902661	0.7720941
LinearFold, MXFold2, ContextFold, CONTRAfold	0.7660585	0.77579325	0.7858325
LinearFold, MXFold2, ContextFold, mfold	0.7678404	0.7793901	0.78182
LinearFold, MXFold2, ContextFold, EternaFold	0.77110124	0.7802511	0.78998876
LinearFold, MXFold2, ContextFold, SPOT-RNA	0.8061688	0.82036203	0.80993104
LinearFold, MXFold2, ContextFold, UFold	0.79965544	0.8276477	0.8060862
LinearFold, MXFold2, CONTRAfold, mfold	0.72079253	0.73120445	0.74365175
LinearFold, MXFold2, CONTRAfold, EternaFold	0.71213883	0.72534066	0.7382383
LinearFold, MXFold2, CONTRAfold, SPOT-RNA	0.7680352	0.7709445	0.7883181
LinearFold, MXFold2, CONTRAfold, UFold	0.74883604	0.7768558	0.77119225
LinearFold, MXFold2, mfold, EternaFold	0.728182	0.7385896	0.7525229
LinearFold, MXFold2, mfold, SPOT-RNA	0.76604784	0.7722306	0.77958274
LinearFold, MXFold2, mfold, UFold	0.7546877	0.7771951	0.772476
LinearFold, MXFold2, EternaFold, SPOT-RNA	0.7727068	0.7753656	0.7908348
LinearFold, MXFold2, EternaFold, UFold	0.7583746	0.77893317	0.78198636
LinearFold, MXFold2, SPOT-RNA, UFold	0.8064375	0.821264	0.81574506
LinearFold, ContextFold, CONTRAfold, mfold	0.72203535	0.7427786	0.73575276
LinearFold, ContextFold, CONTRAfold, EternaFold	0.712172	0.7316832	0.7328283
LinearFold, ContextFold, CONTRAfold, SPOT-RNA	0.7686757	0.78416175	0.781702
LinearFold, ContextFold, CONTRAfold, UFold	0.7492146	0.7910065	0.76569235
LinearFold, ContextFold, mfold, EternaFold	0.729192	0.7502528	0.7441576
LinearFold, ContextFold, mfold, SPOT-RNA	0.773179	0.79471856	0.77729833

LinearFold, ContextFold, mfold, UFold	0.7624328	0.79733396	0.7711952
LinearFold, ContextFold, EternaFold, SPOT-RNA	0.7719309	0.79082656	0.78178906
LinearFold, ContextFold, EternaFold, UFold	0.7599477	0.7946234	0.77482164
LinearFold, ContextFold, SPOT-RNA, UFold	0.8026195	0.8388323	0.7999353
LinearFold, CONTRAfold, mfold, EternaFold	0.69423145	0.7119734	0.716583
LinearFold, CONTRAfold, mfold, SPOT-RNA	0.72554564	0.7400493	0.74233985
LinearFold, CONTRAfold, mfold, UFold	0.71223205	0.7456277	0.72843444
LinearFold, CONTRAfold, EternaFold, SPOT-RNA	0.71474576	0.7310339	0.73747706
LinearFold, CONTRAfold, EternaFold, UFold	0.7077475	0.7317603	0.73016834
LinearFold, CONTRAfold, SPOT-RNA, UFold	0.7608448	0.7847572	0.7848123
LinearFold, mfold, EternaFold, SPOT-RNA	0.729929	0.7453817	0.74570185
LinearFold, mfold, EternaFold, UFold	0.7178708	0.7471309	0.73545384
LinearFold, mfold, SPOT-RNA, UFold	0.7699139	0.796034	0.78288853
LinearFold, EternaFold, SPOT-RNA, UFold	0.76462287	0.7908861	0.78412205
RNAfold, MXFold2, ContextFold, CONTRAfold	0.7632269	0.75582325	0.7973392
RNAfold, MXFold2, ContextFold, mfold	0.7491958	0.73739654	0.7865809
RNAfold, MXFold2, ContextFold, EternaFold	0.7640699	0.7541669	0.8003375
RNAfold, MXFold2, ContextFold, SPOT-RNA	0.7977742	0.79319364	0.81893945
RNAfold, MXFold2, ContextFold, UFold	0.79827774	0.7988923	0.822698
RNAfold, MXFold2, CONTRAfold, mfold	0.7142498	0.71126455	0.7495825
RNAfold, MXFold2, CONTRAfold, EternaFold	0.71791875	0.7150884	0.7593085
RNAfold, MXFold2, CONTRAfold, SPOT-RNA	0.76417416	0.7512436	0.7981218
RNAfold, MXFold2, CONTRAfold, UFold	0.749039	0.7571321	0.7849805
RNAfold, MXFold2, mfold, EternaFold	0.71802217	0.7112772	0.757496
RNAfold, MXFold2, mfold, SPOT-RNA	0.748036	0.7321368	0.7842858
RNAfold, MXFold2, mfold, UFold	0.7378387	0.73773044	0.7758518
RNAfold, MXFold2, EternaFold, SPOT-RNA	0.7642328	0.74963015	0.79844093
RNAfold, MXFold2, EternaFold, UFold	0.7533492	0.7548739	0.79157245
RNAfold, MXFold2, SPOT-RNA, UFold	0.79792786	0.7915022	0.8244824
RNAfold, ContextFold, CONTRAfold, mfold	0.7195137	0.71712095	0.7524179

RNAfold, ContextFold, CONTRAfold, EternaFold	0.7201657	0.72656417	0.7516749
RNAfold, ContextFold, CONTRAfold, SPOT-RNA	0.77201045	0.76873815	0.8005249
RNAfold, ContextFold, CONTRAfold, UFold	0.75849664	0.772459	0.7876994
RNAfold, ContextFold, mfold, EternaFold	0.7216104	0.7180187	0.75580865
RNAfold, ContextFold, mfold, SPOT-RNA	0.7537142	0.7470061	0.7873884
RNAfold, ContextFold, mfold, UFold	0.74741435	0.7464698	0.7847312
RNAfold, ContextFold, EternaFold, SPOT-RNA	0.76932967	0.76888305	0.7964974
RNAfold, ContextFold, EternaFold, UFold	0.7627346	0.7728218	0.7940324
RNAfold, ContextFold, SPOT-RNA, UFold	0.8092589	0.8237864	0.8193058
RNAfold, CONTRAfold, mfold, EternaFold	0.6977667	0.69404775	0.74171704
RNAfold, CONTRAfold, mfold, SPOT-RNA	0.71819454	0.712505	0.75169945
RNAfold, CONTRAfold, mfold, UFold	0.7078193	0.7177043	0.73986435
RNAfold, CONTRAfold, EternaFold, SPOT-RNA	0.7217023	0.72628343	0.7544209
RNAfold, CONTRAfold, EternaFold, UFold	0.7161886	0.72613555	0.7493628
RNAfold, CONTRAfold, SPOT-RNA, UFold	0.76177025	0.7684943	0.79731405
RNAfold, mfold, EternaFold, SPOT-RNA	0.71982914	0.71455497	0.75391656
RNAfold, mfold, EternaFold, UFold	0.7097967	0.7169583	0.7431985
RNAfold, mfold, SPOT-RNA, UFold	0.7508633	0.74527407	0.79435414
RNAfold, EternaFold, SPOT-RNA, UFold	0.7611989	0.7671068	0.7954845
MXFold2, ContextFold, CONTRAfold, mfold	0.7654137	0.75818676	0.7960461
MXFold2, ContextFold, CONTRAfold, EternaFold	0.7607662	0.7501928	0.7975663
MXFold2, ContextFold, CONTRAfold, SPOT-RNA	0.80552775	0.8021358	0.82582504
MXFold2, ContextFold, CONTRAfold, UFold	0.8036846	0.8083685	0.8242811
MXFold2, ContextFold, mfold, EternaFold	0.76975954	0.7608228	0.8001734
MXFold2, ContextFold, mfold, SPOT-RNA	0.7998795	0.79838055	0.81747186
MXFold2, ContextFold, mfold, UFold	0.80149883	0.80406094	0.82156813
MXFold2, ContextFold, EternaFold, SPOT-RNA	0.8055973	0.80042183	0.82592356
MXFold2, ContextFold, EternaFold, UFold	0.80477804	0.8051098	0.8289949
MXFold2, ContextFold, SPOT-RNA, UFold	0.8286949	0.832974	0.8408528
MXFold2, CONTRAfold, mfold, EternaFold	0.7267986	0.7194221	0.7649635

MXFold2, CONTRAfold, mfold, SPOT-RNA	0.7652121	0.7539724	0.79493225
MXFold2, CONTRAfold, mfold, UFold	0.7598695	0.75999224	0.79217696
MXFold2, CONTRAfold, EternaFold, SPOT-RNA	0.7627746	0.74582106	0.79989886
MXFold2, CONTRAfold, EternaFold, UFold	0.7495033	0.74842215	0.7893073
MXFold2, CONTRAfold, SPOT-RNA, UFold	0.8068495	0.8038765	0.83037025
MXFold2, mfold, EternaFold, SPOT-RNA	0.76932234	0.7576637	0.79724437
MXFold2, mfold, EternaFold, UFold	0.7644041	0.76345927	0.7972464
MXFold2, mfold, SPOT-RNA, UFold	0.8009841	0.7981583	0.82287216
MXFold2, EternaFold, SPOT-RNA, UFold	0.80478114	0.800493	0.82782876
ContextFold, CONTRAfold, mfold, EternaFold	0.7272573	0.72722304	0.7575046
ContextFold, CONTRAfold, mfold, SPOT-RNA	0.7717699	0.77018994	0.7972162
ContextFold, CONTRAfold, mfold, UFold	0.7666918	0.7732122	0.7938341
ContextFold, CONTRAfold, EternaFold, SPOT-RNA	0.76285434	0.75536335	0.7963729
ContextFold, CONTRAfold, EternaFold, UFold	0.753348	0.7574194	0.7894058
ContextFold, CONTRAfold, SPOT-RNA, UFold	0.81292737	0.8224295	0.8262646
ContextFold, mfold, EternaFold, SPOT-RNA	0.77437115	0.77562165	0.79658115
ContextFold, mfold, EternaFold, UFold	0.77266294	0.7779622	0.79926586
ContextFold, mfold, SPOT-RNA, UFold	0.8117059	0.82619995	0.8198823
ContextFold, EternaFold, SPOT-RNA, UFold	0.8085416	0.82023954	0.82138956
CONTRAfold, mfold, EternaFold, SPOT-RNA	0.72897655	0.7252587	0.7604535
CONTRAfold, mfold, EternaFold, UFold	0.7241429	0.7281291	0.7559078
CONTRAfold, mfold, SPOT-RNA, UFold	0.77240753	0.7715649	0.80668676
CONTRAfold, EternaFold, SPOT-RNA, UFold	0.7598478	0.75539714	0.8036568
mfold, EternaFold, SPOT-RNA, UFold	0.7727136	0.7757642	0.80256385
LinearFold, RNAfold, MXFold2	0.69196683	0.77193296	0.68628925
LinearFold, RNAfold, ContextFold	0.69276744	0.8020539	0.66726947
LinearFold, RNAfold, CONTRAfold	0.6633242	0.73814607	0.6586835
LinearFold, RNAfold, mfold	0.65299094	0.7256273	0.6514491
LinearFold, RNAfold, EternaFold	0.66879773	0.73814994	0.6667474
LinearFold, RNAfold, SPOT-RNA	0.6928118	0.7941873	0.6704585

LinearFold, RNAfold, UFold	0.67716885	0.8030796	0.65666825
LinearFold, MXFold2, ContextFold	0.7666943	0.83972573	0.74103534
LinearFold, MXFold2, CONTRAfold	0.70151883	0.7709	0.69227564
LinearFold, MXFold2, mfold	0.70958763	0.7795143	0.6945685
LinearFold, MXFold2, EternaFold	0.7135086	0.77843285	0.70647234
LinearFold, MXFold2, SPOT-RNA	0.76920724	0.8305406	0.7468065
LinearFold, MXFold2, UFold	0.725703	0.83965296	0.70783794
LinearFold, ContextFold, CONTRAfold	0.69853896	0.78853875	0.6792923
LinearFold, ContextFold, mfold	0.70851207	0.8049698	0.6777532
LinearFold, ContextFold, EternaFold	0.70932126	0.79694074	0.6886899
LinearFold, ContextFold, SPOT-RNA	0.7622098	0.8543024	0.7244861
LinearFold, ContextFold, UFold	0.73186296	0.8660695	0.69829744
LinearFold, CONTRAfold, mfold	0.670702	0.7422046	0.65992504
LinearFold, CONTRAfold, EternaFold	0.6817339	0.7287429	0.68565875
LinearFold, CONTRAfold, SPOT-RNA	0.7023001	0.78341424	0.6848124
LinearFold, CONTRAfold, UFold	0.67668855	0.78854567	0.660745
LinearFold, mfold, EternaFold	0.67461044	0.7460834	0.66530323
LinearFold, mfold, SPOT-RNA	0.7106639	0.8025144	0.6810795
LinearFold, mfold, UFold	0.67876935	0.8074754	0.6528459
LinearFold, EternaFold, SPOT-RNA	0.70881605	0.7942763	0.690181
LinearFold, EternaFold, UFold	0.68466526	0.7980608	0.6684153
LinearFold, SPOT-RNA, UFold	0.7329488	0.8526088	0.70875734
RNAfold, MXFold2, ContextFold	0.77113706	0.80322045	0.76666355
RNAfold, MXFold2, CONTRAfold	0.71355504	0.75032324	0.7200887
RNAfold, MXFold2, mfold	0.69497055	0.72655946	0.70132744
RNAfold, MXFold2, EternaFold	0.7190875	0.74626845	0.72940165
RNAfold, MXFold2, SPOT-RNA	0.76844907	0.7947543	0.7648724
RNAfold, MXFold2, UFold	0.74293214	0.8028096	0.7426626
RNAfold, ContextFold, CONTRAfold	0.7186833	0.76765406	0.71497434
RNAfold, ContextFold, mfold	0.69936377	0.73650116	0.69795877

RNAfold, ContextFold, EternaFold	0.7232984	0.7658697	0.7224107
RNAfold, ContextFold, SPOT-RNA	0.78195935	0.84041196	0.75842595
RNAfold, ContextFold, UFold	0.7578029	0.8417937	0.7375031
RNAfold, CONTRAfold, mfold	0.6773142	0.7102866	0.6848001
RNAfold, CONTRAfold, EternaFold	0.69054824	0.72126865	0.7045376
RNAfold, CONTRAfold, SPOT-RNA	0.71760964	0.7624389	0.7156616
RNAfold, CONTRAfold, UFold	0.7088692	0.7707856	0.70666325
RNAfold, mfold, EternaFold	0.6780974	0.7089205	0.6868479
RNAfold, mfold, SPOT-RNA	0.6929759	0.7299946	0.69075555
RNAfold, mfold, UFold	0.67792875	0.73501074	0.6782618
RNAfold, EternaFold, SPOT-RNA	0.7172642	0.763551	0.7156022
RNAfold, EternaFold, UFold	0.7090512	0.76425385	0.70961463
RNAfold, SPOT-RNA, UFold	0.7580661	0.8360476	0.74425524
MXFold2, ContextFold, CONTRAfold	0.7803103	0.8135475	0.77413636
MXFold2, ContextFold, mfold	0.77382547	0.8091433	0.7634771
MXFold2, ContextFold, EternaFold	0.7821988	0.81209147	0.77826494
MXFold2, ContextFold, SPOT-RNA	0.81038505	0.8495949	0.792889
MXFold2, ContextFold, UFold	0.80345213	0.85638976	0.7863521
MXFold2, CONTRAfold, mfold	0.72387266	0.7530758	0.7261649
MXFold2, CONTRAfold, EternaFold	0.7159021	0.7389697	0.7296681
MXFold2, CONTRAfold, SPOT-RNA	0.7804832	0.8095525	0.7749847
MXFold2, CONTRAfold, UFold	0.76494795	0.81728095	0.76151806
MXFold2, mfold, EternaFold	0.7324801	0.76066583	0.7370554
MXFold2, mfold, SPOT-RNA	0.7689416	0.8016245	0.7587225
MXFold2, mfold, UFold	0.75792956	0.80797595	0.75162023
MXFold2, EternaFold, SPOT-RNA	0.77919173	0.80603456	0.7746357
MXFold2, EternaFold, UFold	0.7692489	0.81181943	0.7695874
MXFold2, SPOT-RNA, UFold	0.80460876	0.84724325	0.7914163
ContextFold, CONTRAfold, mfold	0.72562885	0.7687969	0.7173603
ContextFold, CONTRAfold, EternaFold	0.7167161	0.7476334	0.7242585

ContextFold, CONTRAfold, SPOT-RNA	0.78531224	0.8364876	0.76522774
ContextFold, CONTRAfold, UFold	0.7669109	0.84526175	0.74719524
ContextFold, mfold, EternaFold	0.73348355	0.7766108	0.72626346
ContextFold, mfold, SPOT-RNA	0.78178114	0.84126395	0.7548336
ContextFold, mfold, UFold	0.76915157	0.84291136	0.74506545
ContextFold, EternaFold, SPOT-RNA	0.78161055	0.8353781	0.76085263
ContextFold, EternaFold, UFold	0.7718151	0.83995605	0.75306314
ContextFold, SPOT-RNA, UFold	0.79703426	0.8674035	0.7697988
CONTRAfold, mfold, EternaFold	0.6944757	0.72326744	0.7037904
CONTRAfold, mfold, SPOT-RNA	0.7253816	0.7652684	0.7191091
CONTRAfold, mfold, UFold	0.7142934	0.7726327	0.7074936
CONTRAfold, EternaFold, SPOT-RNA	0.7144456	0.7449742	0.72200584
CONTRAfold, EternaFold, UFold	0.709931	0.7468132	0.7177114
CONTRAfold, SPOT-RNA, UFold	0.77416265	0.8368357	0.76270294
mfold, EternaFold, SPOT-RNA	0.73030585	0.770563	0.7235628
mfold, EternaFold, UFold	0.7204792	0.7746157	0.7154551
mfold, SPOT-RNA, UFold	0.7739969	0.84082097	0.7559088
EternaFold, SPOT-RNA, UFold	0.77285653	0.8335128	0.7609427
LinearFold, RNAfold	0.65308607	0.6488183	0.71691066
LinearFold, MXFold2	0.7508829	0.71358836	0.81508034
LinearFold, ContextFold	0.7498478	0.72192615	0.80468166
LinearFold, CONTRAfold	0.6802448	0.6804915	0.7277456
LinearFold, mfold	0.6646309	0.6311337	0.725356
LinearFold, EternaFold	0.6820812	0.67516357	0.7365612
LinearFold, SPOT-RNA	0.74785984	0.71698034	0.80766696
LinearFold, UFold	0.707786	0.71475685	0.77983785
RNAfold, MXFold2	0.7305297	0.68027526	0.8181209
RNAfold, ContextFold	0.73388356	0.6749569	0.83738804
RNAfold, CONTRAfold	0.6758101	0.6484038	0.7634108
RNAfold, mfold	0.6583778	0.61962324	0.72524035

RNAfold, EternaFold	0.6769485	0.6482043	0.7612178
RNAfold, SPOT-RNA	0.7258365	0.66574746	0.82811713
RNAfold, UFold	0.7059488	0.6671441	0.8178657
MXFold2, ContextFold	0.7898312	0.7430582	0.8633778
MXFold2, CONTRAfold	0.7491312	0.6940393	0.8373138
MXFold2, mfold	0.7234935	0.66723627	0.8173008
MXFold2, EternaFold	0.7464862	0.6962657	0.83055425
MXFold2, SPOT-RNA	0.78266263	0.7320792	0.8573421
MXFold2, UFold	0.7915329	0.7384756	0.8772144
ContextFold, CONTRAfold	0.75176436	0.6952965	0.84209996
ContextFold, mfold	0.73050475	0.67092884	0.8318335
ContextFold, EternaFold	0.7513161	0.69843787	0.8403557
ContextFold, SPOT-RNA	0.79944706	0.75430024	0.863134
ContextFold, UFold	0.79092056	0.7505002	0.8599395
CONTRAfold, mfold	0.68176675	0.62793046	0.76785225
CONTRAfold, EternaFold	0.69491565	0.6753797	0.75737107
CONTRAfold, SPOT-RNA	0.7483553	0.69191545	0.84031916
CONTRAfold, UFold	0.7360359	0.6924611	0.84031576
mfold, EternaFold	0.68288153	0.6297716	0.77205515
mfold, SPOT-RNA	0.7209696	0.65621084	0.82455426
mfold, UFold	0.7204264	0.6569807	0.8311517
EternaFold, SPOT-RNA	0.74418604	0.6891925	0.8319003
EternaFold, UFold	0.7356645	0.6920189	0.8359937
SPOT-RNA, UFold	0.77752423	0.7412249	0.837066
LinearFold	0.5771588	0.7375111	0.55931085
RNAfold	0.64335907	0.66150796	0.6690368
MXFold2	0.77388155	0.76644504	0.79160374
ContextFold	0.7733564	0.7959753	0.76084936
CONTRAfold	0.6858665	0.70063543	0.70967424
mfold	0.6454754	0.6401375	0.66021913

EternaFold	0.6880218	0.69982845	0.7122904
SPOT-RNA	0.7295961	0.7365224	0.75404394
UFold	0.6178378	0.72721475	0.62271565
