# Supplementary material for: Enhanced Generalizability of RNA Secondary Structure Prediction via Convolutional Block Attention Network and Ensemble Learning
Source: Molecules. 2025 Aug 21;30(16):3447. doi: 10.3390/molecules30163447 (PMC12388828; doi:10.3390/molecules30163447)

Supplementary Material

Enhanced generalizability of RNA secondary structure prediction via convolutional block attention network and ensemble learning

Hanbo Lin[1,#], Dongyue Hou[1,#], Zhaoyite Li[2], Shuaiqi Wang[1], Yuchen Liu[1], Jiajie Gu[1], Juncheng Qian[1], Ruining Yin[1], Hui Zhao[3], Shaofei Wang[4], Yuzong Chen[5,], Dianwen Ju[1,*], Xian Zeng[1,*]*

# These authors contributed equally to this work.

[1] School of Pharmaceutical Sciences, Shanghai Engineering Research Center of Immunotherapeutics, Fudan University, Shanghai 201203, China;

[2] The State Key Laboratory of Chemical Oncogenomics, Key Laboratory of Chemical Biology, Tsinghua Shenzhen International Graduate School, Tsinghua University, Shenzhen, 518055, P. R. China;

[3] Byterna therapeutics Ltd., Shanghai 201203, P.R. China;

[4] Department of Cellular and Genetic Medicine, School of Basic Medical Sciences, Fudan University, Shanghai 200032, P.R. China;

[5] Institute of Biomedical Health Technology and Engineering, Shenzhen Bay Laboratory, Shenzhen 518000, P. R. China.

Table S2. Distribution of maximum sequence identity between TestSetA dataset and training sets

Identity range (%)	Proportion of TestSetA dataset (%)
90-100	27.96
80-90	3.76
70-80	6.63
60-70	4.48
50-60	6.09
40-50	4.30
30-40	0
<30	46.77

Table S3. Distribution of maximum sequence identity between TS0 dataset and training sets

Identity range (%)	Proportion of TS0 dataset (%)
90-100	11.27
80-90	1.94
70-80	4.12
60-70	2.25
50-60	1.71
40-50	2.64
30-40	0
<30	76.07

Table S4. Average inference time for sequences in TestSetA across different algorithms.

Algorithm	Inference time (s)
UFold (GPU)	0.0261
SPOT-RNA (GPU)	3.5529
ContextFold	0.4416
EternaFold	0.3425
RNAfold	0.1760
LinearFold	0.1241
CONTRAfold	0.0649
MXFold2	2.4818
TrioFold (GPU)	0.0842

Table S5. Distribution of maximum sequence identity between bpRNA-new dataset and training sets

Identity range (%)	Proportion of bpRNA-new dataset (%)
90-100	0
80-90	0
70-80	0.02
60-70	0.02
50-60	0.72
40-50	2.95
30-40	0.02
<30	96.27

Table S6. Distribution of maximum sequence identity between PDB dataset and training sets

Identity range (%)	Proportion of PDB dataset (%)
90-100	8.40
80-90	1.68
70-80	5.04
60-70	0.84
50-60	2.52
40-50	0.84
30-40	0
<30	80.67

Table S7. Comparative evaluation of pseudoknot prediction on PDB dataset

Algotirhm	Precision	Recall	F1 score
TrioFold	0.109	0.055	0.068
SPOT-RNA	0.120	0.077	0.089
UFold	0.228	0.154	0.174

Table S8. The comparison of algorithms' parameters

Algorithm	Parameters
UFold	8641377
SPOT-RNA	7759445
ContextFold	~70000
E2Efold	718863
MXfold2	47436
TrioFold	2837

Supplementary Figure S1.

Line chart showing algorithm performance based on dataset sequences filtered at different similarity cutoffs.

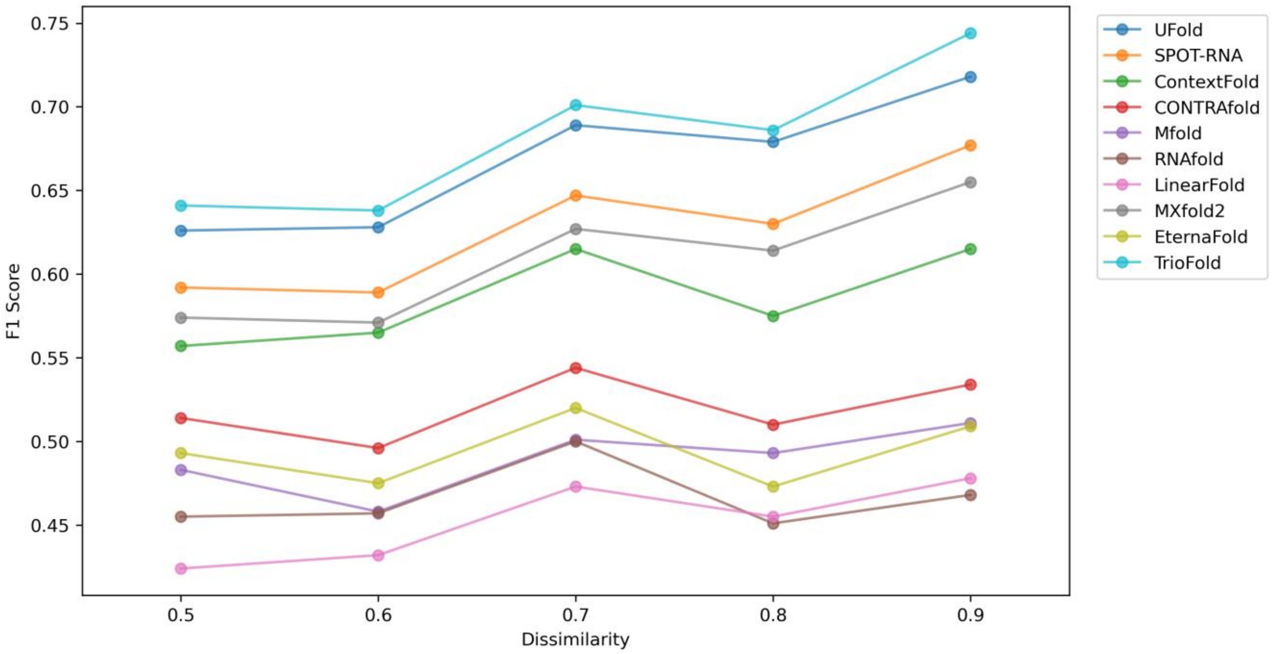

Supplementary Figure S2.

Jaccard distance map of secondary structure predictions from different algorithms. A higher distance indicates greater differences between the predicted structures of the two algorithms.

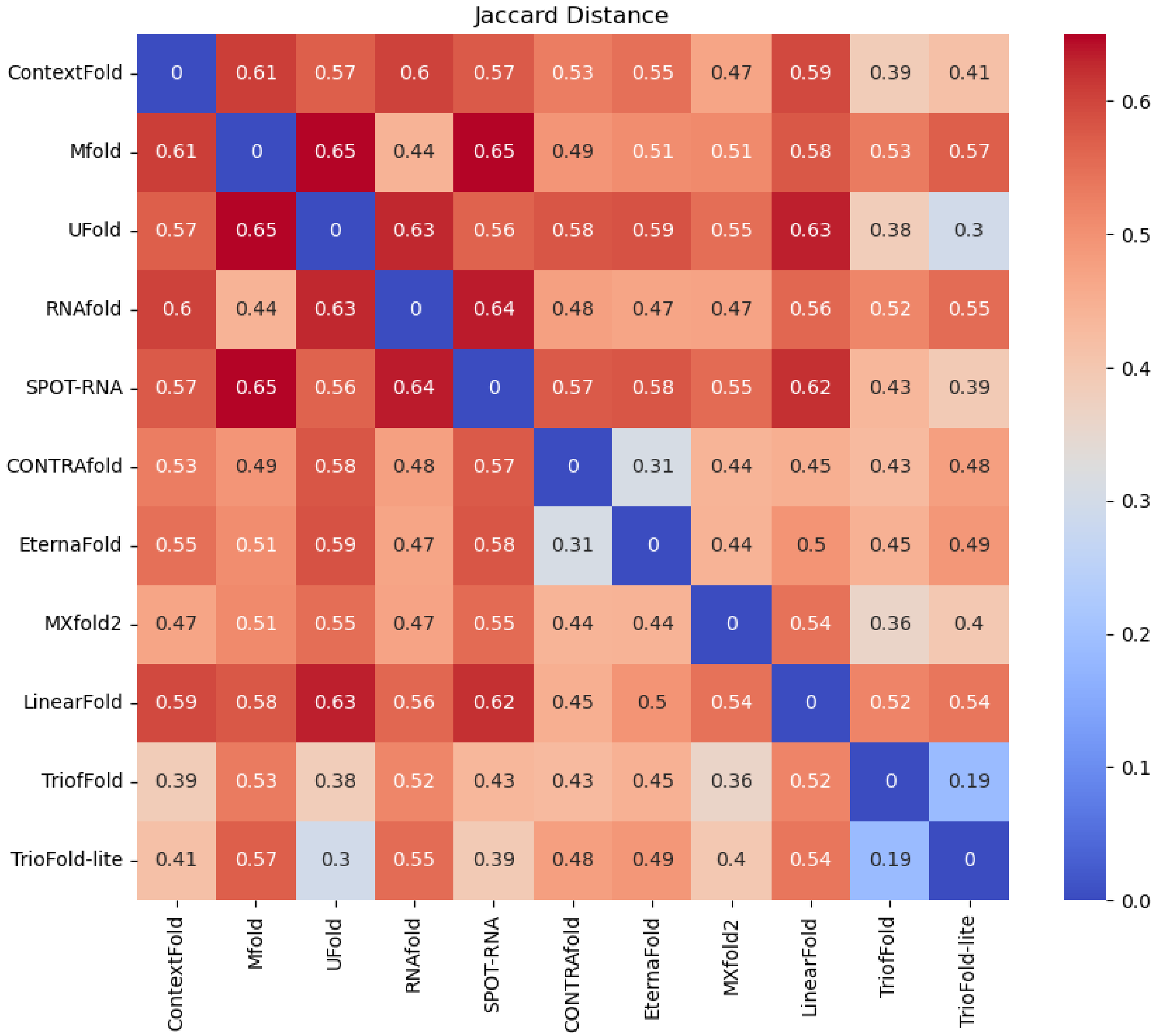

Supplementary Figure S3.

Box plot with scatter points illustrating the Jaccard distances between a specific algorithm and others. A higher median in the box plot indicates greater divergence of the algorithm from the others.

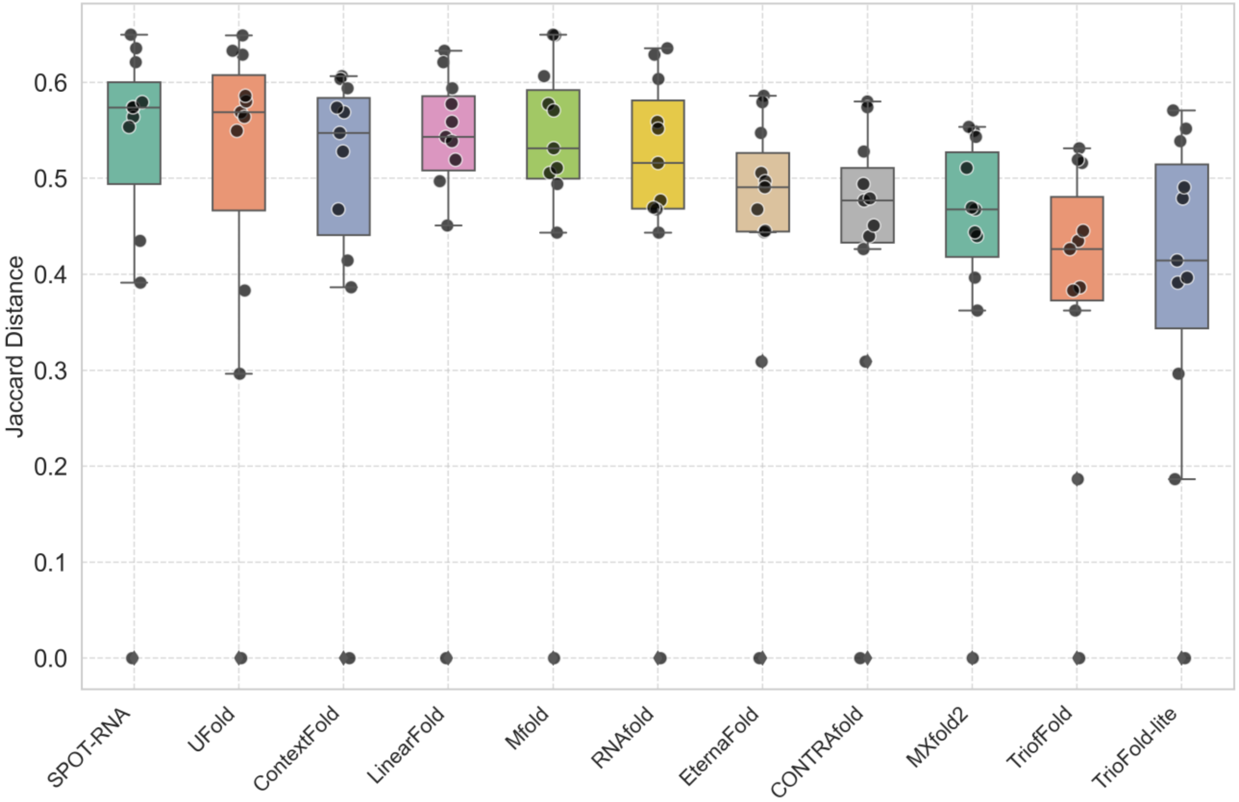

Supplement: Supplementary file 1 [file molecules-30-03447-s001.zip › Tables S2-S8, Figures S1-S3.pdf]
